# Risk of SARS-CoV-2 infection in migrants and ethnic minorities compared with the general population in the European WHO region during the first year of the pandemic: a systematic review

**DOI:** 10.1186/s12889-021-12466-1

**Published:** 2022-01-20

**Authors:** Anissa Jaljaa, Susanna Caminada, Maria Elena Tosti, Franca D’Angelo, Aurora Angelozzi, Claudia Isonne, Giulia Marchetti, Elena Mazzalai, Dara Giannini, Federica Turatto, Chiara De Marchi, Angela Gatta, Silvia Declich, Scilla Pizzarelli, Salvatore Geraci, Giovanni Baglio, Maurizio Marceca

**Affiliations:** 1 Department of Public Health and Infectious Diseases, Sapienza University, Rome, Italy; 2 Italian Society of Migration Medicine (SIMM), Rome, Italy; 3 National Health Institute, National Centre for Global Health, Rome, Italy; 4 National Health Institute; Knowledge Service, Documentation and Library, Rome, Italy; 5 Caritas of Rome, Health Area, Rome, Italy; 6 AGENAS, Research and International Relations Office, Rome, Italy

**Keywords:** Migrants, Ethnic minorities, SARS-CoV-2 infection, COVID-19, Systematic review, Health inequalities

## Abstract

**Background:**

Migrants and ethnic minorities have suffered a disproportionate impact of the COVID-19 pandemic compared to the general population from different perspectives. Our aim was to assess specifically their risk of infection in the 53 countries belonging to the World Health Organization European Region, during the first year of the pandemic.

**Methods:**

We conducted a systematic review following Preferred Reporting Items for Systematic Reviews and Meta-Analyses guidelines (PROSPERO CRD42021247326). We searched multiple databases for peer-reviewed literature, published on Medline, Embase, Scisearch, Biosis and Esbiobase in 2020 and preprints from PubMed up to 29/03/2021. We included cross-sectional, case-control, cohort, intervention, case-series, prevalence or ecological studies, reporting the risk of SARS-CoV-2 infection among migrants, refugees, and ethnic minorities.

**Results:**

Among the 1905 records screened, 25 met our inclusion criteria and were included in the final analysis. We found that migrants and ethnic minorities during the first wave of the pandemic were at increased exposure and risk of infection and were disproportionately represented among COVID-19 cases. However, the impact of COVID-19 on minorities does not seem homogeneous, since some ethnic groups seem to be more at risk than others. Risk factors include high-risk occupations, overcrowded accommodations, geographic distribution, social deprivation, barriers to access to information concerning preventive measures (due to the language barrier or to their marginality), together with biological and genetic susceptibilities.

**Conclusions:**

Although mixed methods studies will be required to fully understand the complex interplay between the various biological, social, and cultural factors underlying these findings, the impact of structural determinants of health is evident. Our findings corroborate the need to collect migration and ethnicity-disaggregated data and contribute to advocacy for inclusive policies and programmatic actions tailored to reach migrants and ethnic minorities.

**Supplementary Information:**

The online version contains supplementary material available at 10.1186/s12889-021-12466-1.

## Background

With more than 175 million infected globally and more than 3.8 million deaths at the time of writing [1, 2] the COVID-19 pandemic stands as the greatest public health challenge since the 1918 influenza pandemic [3], urgently raising the need to ensure the protection of health for all [4]. The scientific community has emphasized how the effects of the pandemic are contributing to increasing health inequalities, through direct mechanisms - which concern exposure to infection risk and vulnerability - and indirect mechanisms, due to the impact of the pandemic on the organization of the Health System and on the socio-economic determinants of health [5].

Migrants and Ethnic Minorities (MEMs) generally represent a potentially vulnerable population group [6, 7], in part because they are more frequently subject to discrimination and socio-economic inequalities [8, 9]. In the context of the current pandemic, it is therefore increasingly necessary to pay special attention to these populations [10–12].

During the last year, the scientific literature regarding Severe Acute Respiratory Syndrome Coronavirus-2 (SARS-CoV-2) has grown enormously, involving the entire scientific community at a global level [13]. Nowadays, the PubMed database counts more than 140,890 articles related to COVID-19 [14]. Among the published studies, there are several on the epidemiological and clinical characteristics of at-risk population groups, which have revealed that ethnic minorities have a higher risk of getting sick and dying [15–17]. However, the available studies do not offer, until now, unambiguous interpretive models about the risk of infection among different population groups and the dynamics that sustain it, especially in the World Health Organization (WHO) European region.

This is the first study to date that aims to assess, through a systematic review of the literature, what has been the risk of infection with SARS-CoV-2 virus for migrants and ethnic minorities in the 53 countries belonging to the European Region of the WHO [18] and whether this differs significantly from the risk of infection of the corresponding autochthonous populations. In addition, the study aims at suggesting possible explanations based on the findings of the included studies and literature. Further studies on the risk of mortality, other clinical outcomes, and differences in access to services are ongoing in the context of a wider research project.

## Methods

A systematic review was undertaken according to the Preferred Reporting Items for Systematic Reviews and Meta-Analyses (PRISMA) [19] in order to retrieve studies evaluating the exposure to SARS-CoV-2 of migrants, refugees and ethnic minorities. It was registered with PROSPERO (CRD42021247326). A protocol for this systematic review was prepared and published on PROSPERO.

We used the STN® international platform to search the following databases: Medline, Embase, Scisearch, Biosis, Esbiobase. To increase efficiency all databases were searched simultaneously with a single query.

After a preliminary pilot search aiming at balancing recall (sensitivity) and precision (specificity), a tailored search strategy was developed. It included both Medline and Embase subject headings (i.e. MeSH and Emtree terms, respectively) as well as free-text words in the title and abstract fields. Duplicate citations due to databases overlap were removed during the multifile session and de-duplicated search results were exported to Microsoft Excel to facilitate further data analysis and management. Further duplicates, not automatically intercepted, were removed based on a review of the titles.

In order to identify emergent literature on our topic, an additional search for preprint citations was run in PubMed, which makes available content from the following preprint servers: medRxiv, bioRxiv, ChemRxiv, arXiv, Research Square, and Social Science Research Network.

The complete search strategies are presented as an additional file (see Additional file 1).

### Selection criteria

We included studies reporting the risk of SARS-CoV-2 infection among migrants, refugees, and ethnic minorities in the 53 countries belonging to the European Region of WHO. For migrant definition we referred to the International Organization for Migration (IOM) glossary^1^ [20]. We sourced refugee definition from the convention and protocol relating to the status of refugees of the United Nations High Commissioner for Refugees^2^ [21]. At last, we defined ‘ethnic minorities’ according to the European Centre for Disease Prevention and Control^3^ [22]. However, it must be noted that there is no universally accepted definition of such terms and that the differences between migrants and ethnic minorities are nuanced and dependent on the country context.

We included primary quantitative and quali-quantitative studies (cross-sectional, case-control, cohort, intervention, case-series, prevalence or ecological studies), published in English, Italian, French and Spanish languages, in 2020 on the databases cited above, purely qualitative studies were excluded. Regarding the publication type, comments, opinions, editorials, and news were excluded; letters were included only if containing original quantitative data. Reviews were excluded, but if they were relevant to our topic, their sources (primary studies) were included. The preprints were searched on PubMed up to March 29, 2021.

Two reviewers at a time screened independently title and abstract against eligibility criteria. Overall, ten researchers, appropriately trained and constantly monitored, were involved. Furthermore, by reading the full texts, they assessed eligibility for inclusion of the selected studies. Any disagreements were resolved by discussion between the two reviewers. If it was not possible, an assessment group stepped in for a final decision.

### Critical appraisal, data extraction and synthesis

Two researchers assessed the study quality independently, using the appropriate Joanna Briggs Institute critical appraisal tools [23] for each study design. Quality scores were calculated as the number of positive answers out of the maximum number of applicable questions and converted into percentages. Studies with a score of 80–100% were considered ‘high quality’, 60–79% ‘medium quality’ and 0–59% ‘low quality’ [24]. Low quality studies were not excluded but contributed to the final synthesis. For each selected study, the study quality in terms of risk of bias is presented in the Results.

Relevant information from the included documents was extracted by one reviewer and checked by another, using an appropriate extraction form that included the following items: bibliographic reference, publication country, language, type of study, study period, objectives, exposed population, comparison population if available, diagnostic methods, observation setting, outcomes and their measures of effect (in terms of incidence, prevalence, morbidity rates, rate ratios, odds ratios, relative risks, hazard ratios), results, conclusions, limits, comments and study quality.

Disagreements both in quality assessment and in data extraction were resolved by discussion between the two reviewers. If it was not possible, an assessment group stepped in for a final decision.

Data of the included studies were narratively described and gathered according to outcomes and effect measures and illustrated in a table. Due to heterogeneity of study designs and populations, meta-analysis was not performed.

## Results

### Literature search and selection

**Fig. 1:**
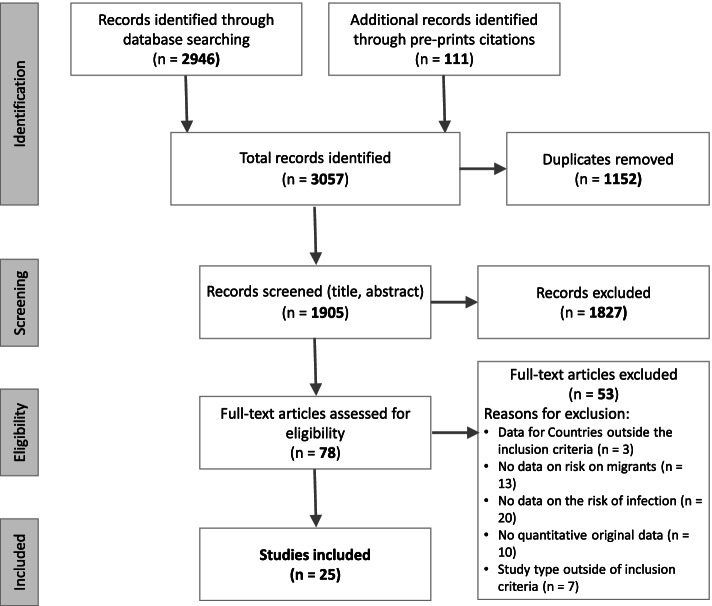
PRISMA flow diagram of included data sources

The systematic search of the literature concerning the study question identified 2946 records on databases and 111 records on preprint citations; 1152 duplicates were removed, so 1905 records were screened by title and abstract and then the remaining 78 by full text reading. Twenty-five records, all peer reviewed, met all the inclusion criteria and were analysed for a quality appraisal (Fig. 1), no preprint was included.

Sixty-eight percent of studies were considered “high quality”, 28% “medium quality” and only one study had a “low quality” level. Table 1 shows the characteristics of the included studies. For further details on the included studies see Additional file 2.

**Table 1:** Characteristics of the included studies

Citation	Location, Period	Study design	Population (Subgroups)	Sample size	Outcome	Effect measures	Study quality
Guijarro C., October 2020 [25]	Spain February 1 – April 25, 2020	Population-based cohort study	Migrants	152,018	Cumulative incidence of RT-PCR^b^ confirmed COVID-19	Cumulative incidence, Relative Risk	High
Jaqueti Aroca J., May 2020 [26]	Spain Up to the 2nd week of April 2020	Cross-sectional	Migrants	1781	Positive SARS-CoV-2 RT-PCR^b^	Positivity Rates	Medium
Amengual-Moreno M., September 2020 [27]	Spain Until May 14, 2020	Observational retrospective ecological study	Migrants	–	Correlation of the cumulative incidence confirmed by PCR^b^ with percentage of immigration of the neighbourhood	r (Pearson linear correlation coefficient)	High
Burton-Jeangros C., December 2020 [28]	Switzerland April – May 2020	Cross-sectional mixed-methods study	Migrants	379	Referred COVID-19 infection in a questionnaire	Prevalence of reported infection	Medium
Lundkvist A., August 2020 [29]	Sweden June 17–18, 2020	Cross-sectional study	Non-Swedish individuals	213	Positive tests for SARS-CoV-2 antibodies	Prevalence	Medium
The Reggio Emilia COVID-19 Working Group, August 2020 [30]	Italy March 6 – March 26, 2020	Prevalence study	Migrants	2635	Positive SARS-CoV-2 PCR^b^	Prevalence, Odds Ratio	High
Woolford S.J., July 2020 [32]	UK March 16 – June 1, 2020	Prevalence study	BAME^a^	502,640	Positive SARS-CoV-2 PCR^b^	Prevalence	Medium
Raisi-Estabragh Z., August 2020 [33]	UK March 16 – May 18, 2020	Prospective cohort study	BAME^a^	4510	COVID-19 positive test	Incidence Rates	High
Kolin D.A., November 2020 [36]	UK From March 16, 2020	Prospective cohort study	BAME^a^	397,064	Positive SARS-CoV-2 PCR^b^	Relative Risk, Odds ratio	High
Niedzwiedz C.L., May 2020 [37]	UK March 16 – May 3, 2020	Prospective cohort study	Ethnic minorities	2658	Positive SARS-CoV-2 microbiological tests	Relative Risk	High
Chadeau-Hyam M., October 2020 [38]	UK March 16 – May 18, 2020	Prospective cohort study	Ethnic minorities	488,083	Positive SARS-CoV-2 RT-PCR^b^	Odds Ratio	High
Razieh C., October 2020 [39]	UK March 16 – June 14, 2020	Prevalence study	BAME^a^	5623	Interaction between ethnicity and obesity on the risk of COVID-19 positive laboratory test	Prevalence, Odds Ratio stratified by Body Mass Index	Medium
Kakkar N., May 2020 [40]	UK March 1 – April 25, 2020	Cross-sectional study	BAME^a^	3018	Positive COVID-19 tests	Prevalence	Low
de Lusignan S., May 2020 [41]	UK January 28 – April 4, 2020	Cross-sectional study	Ethnic minorities	3802	Positive SARS-CoV-2 RT-PCR^b^	Odds Ratio	High
Hull A.S., October 2020 [42]	UK January 1 – April 30, 2020	Cross-sectional study	Ethnic minorities	1,257,137	The diagnosis of suspected COVID-19 based on contact history and symptoms given by patients	Prevalence, Odds Ratio	High
Nguyen L.H., July 2020 [43]	UK, USA March – April, 2020	Prospective cohort study	Ethnic minorities (General population; Frontline healthcare workers)	2,035,395 99,795	Report of a positive COVID-19 test through an app	Hazard Ratio	High
Martin C.A., July 2020 [44]	UK March 1 – April 28, 2020	Retrospective cohort study	Ethnic minorities	4051	Positive SARS-CoV-2 PCR^b^ before/after lockdown	Positivity Rates, Odds Ratio	High
Platt L., June 2020 [46]	UK Up to May 2020	Ecological study	Ethnic minorities	–	Predicted number of lab-confirmed COVID-19 cases per 100,000 of group population	Incidence rates	Medium
Birenbaum-Carmeli D., September 2020 [47]	Israel February 23 – June 2, 2020	Cross-sectional study	Ethnic minorities	–	Number of confirmed COVID-19 cases in each community	Morbidity rates	High
Shields A., September 2020 [48]	UK April 24–25, 2020	Cross-sectional study	BAME^a^ (Healthcare workers)	516	Positive tests for SARS-CoV-2 antibodies	Prevalence, Odds Ratio	High
Martin C.A., November 2020 [49]	UK May 2020	Cross-sectional study	Ethnic minorities (Hospital staff members)	10,662	Positive tests for SARS-CoV-2 antibodies	Prevalence, Odds Ratio	High
Razvi S., November 2020 [50]	UK May 28 – June 8, 2020	Cross-sectional study	BAME^a^ (Healthcare workers)	2100	Positive tests for SARS-CoV-2 antibodies	Prevalence, Odds ratio	High
Leeds J.S., August 2020 [51]	UK April 1–28, 2020	Cohort study	BAME^a^ (Healthcare workers)	991	Positive SARS-CoV-2 RT-PCR^b^	Incidence Rates, Odds Ratio	High
Knight M., June 2020 [52]	UK March 1 – April 14, 2020	Prospective population-based cohort study	Ethnic minorities (Pregnant women)	427	Cumulative incidence of PCR^b^ confirmed COVID-19	Incidence Rates, Rate Ratio	Medium
Elias M., October 2020 [53]	France March 1 – April 30, 2020	Prospective cohort study	Ethnic minorities (Kidney recipients)	1216	Positive SARS-CoV-2 PCR^b^	Odds Ratio	High

^a^ BAME: Black, Asian and Minority Ethnicity

^b^ PCR: Polymerase Chain Reaction; RT-PCR: Reverse Transcription Polymerase Chain Reaction

No studies specifically concerning refugees were found. Given the non-homogeneity of the definitions of migrants or ethnic minorities in the different countries, we will quote the term used by the authors of each article.

### Migrants

We found 6 data sources reporting the incidence of COVID-19 specifically in migrants, including 3 from Spain, 1 from Italy, 1 from Switzerland and 1 from Sweden.

In a cohort study conducted in Alcorcón (Spain), involving 152,018 individuals up to 25th April 2020, the crude cumulative incidence rate of COVID-19 among migrants was higher than among the host Spanish population, at 8.71 and 6.51 per 1000 inhabitants respectively (*p* < 0.001) [25]. A markedly increased relative risk (RR) was found in people from Sub-Saharan Africa (RR 3.66, 95% CI [1.42–9.41], *p* = 0.007), the Caribbean (RR 6.35, 95% CI [3.83–10.55], p < 0.001), and Latin America (RR 6.92, 95% CI [4.49–10.67], p < 0.001). Data from a hospital in Madrid [26] showed no significant differences between migrants and host population in terms of COVID-19 positivity among those tested (52.5% [136/259] vs 51.4% [782/1522]) up to the second week of April. There was also no difference in testing rate in terms of odds ratio (OR) between migrants and the host population (OR 1.08, 95% CI [0.95–1.24]). However, positivity rates per 1000 people were higher for migrants from Latin America, in particular Peru (29.6, 95% CI [18-41]), Ecuador (28.7, 95% CI [18-39]), Colombia (11.9, 95% CI [6-17]) and Venezuela (9.9, 95% CI [2-18]), compared with the host population (4.5, 95% CI [4-5]) and other migrant groups. Only 12.5% of COVID-19 positive migrants were older than 65 years of age, compared to 56.9% of Spanish citizens who tested positive. In an ecological study from Barcelona [27] there was a statistically significant negative correlation between the cumulative incidence of COVID-19 up to 14 May 2020 and the percentage of immigration in the neighbourhood (r = − 0.257; *p* < 0.05). However, once more, the percentage of Latin American immigrants in the neighbourhood showed a statistically significant positive correlation (r = 0.322; *p* < 0.01).

A Swiss study [28], with a sample size of 379, found that during the first wave, in Geneva, undocumented and recently regularized migrants frequently reported having had COVID-19 infection: 12.4% of the interviewees (mainly middle-aged women from Latin-America), with no significant difference between the regularized group (10.5%) and the undocumented (16.7%).

A Swedish study reported that, in June 2020, there was a different antibody prevalence rate between two areas of Stockholm with different presence of non-Swedish origin individuals (*N* = 213): in one suburban area, with 98.9% of people of non-Swedish origin among tested individuals, there was a 30% positivity, while in the compared area, with 98.4% of Swedish among tested individuals, the prevalence was 4.1% (*p* < 0.001) [29].

A study conducted in Reggio Emilia (Italy) among 2635 individuals, however, found that immigrant residents and Italians had a similar prevalence of COVID-19 in March 2020 [30]. The probability of being positive among those tested was in fact similar between immigrants and Italians (OR 1.1, 95% CI [0.83–1.5]), as well as the proportion of tested people among foreigners and Italians (OR 0.93, 95% CI [0.81–1.1]). Furthermore, the adjusted ORs confirmed that the probability of testing positive was higher in immigrant women (OR 2.7, 95% CI [1.4–4.9]) than in immigrant men, while in Italians the opposite was found (OR 0.74, 95% CI [0.62–0.88]).

### Ethnic minorities

We found 19 data sources reporting the incidence of COVID-19 in ethnic minorities, including 17 from the United Kingdom (UK), 1 from France and 1 from Israel. 12 concerned the general population, 5 focused on healthcare workers, while 2 dealt with specific vulnerable subgroups.

Out of the 17 studies conducted in the UK, 6 used as data source the UK Biobank cohort, which is a prospective cohort study of over half a million men and women aged 40–69 years old [31]. Several of these studies pointed out that, among the COVID-19 positive cases identified in the first wave, there was an overrepresentation of BAME (Black, Asian, and Minority Ethnic) individuals. A prevalence study reported that non-White individuals were 13.8% of the COVID-19 positive cohort and 7.9% of the negative one (*p* < 0.001) [32]. A prospective cohort study (*N* = 4510) confirmed that BAME ethnicity was associated with greater odds of COVID-19 positive status (OR 1.78, 95% CI [1.43–2.20]), and added that individuals of Black and Asian ethnicity were most disproportionately affected, with Black ethnicities contributing over 3.5 times the number of positive cases (among the 13.1% of positive cases that were BAME individuals, 5.7% were Black, 4.5% Asian; 3.8%) [33]. In another study it was reported that the unadjusted relative risk of COVID-19 for Black participants to the UK Biobank cohort was 3.66 (95% CI [2.83–4.74]), compared to White participants, but adjusting for Townsend deprivation index [34, 35] reduced the relative risk to 2.44 (95% CI [1.86–3.20]) [36]. Another study including 2658 individuals out of the UK Biobank cohort, confirmed that Black and South Asian participants had the highest risk (respectively RR 3.35, 95%CI [2.48–4.53] and RR 2.42, 95% CI [1.75–3.36]), with an attenuation of the risk with adjustment for the country of birth (RR 3.13, 95% CI [2.18–4.48]), for a history of being a healthcare worker (RR 2.66, 95% CI [1.83–3.84]), and for other social factors including employment status, housing tenure and household size (RR 2.05, 95% CI [1.39–3.03]). Among south Asians, risks were largest in particular in the Pakistani group (RR 3.24, 95% CI [1.73–6.07]), and were slightly higher in the Indian group (RR 1.98, 95% CI [1.26–3.09]) [37]. A further cohort study also reported that non-White ethnicity is associated with increased risk of testing positive for SARS-CoV-2 (OR 2.14, 95% CI [1.57–2.93] for Blacks and OR 1.68, 95% CI [1.29–2.18] for other ethnicities), but the risk is attenuated in a fully adjusted complex model for demographic descriptors, social, health risk, medical, and environmental factors (OR 1.11, 95% CI [1.03–1.19] for Blacks and OR 1.14, 95% CI [1.06–1.23] for other ethnicities) [38]. A prevalence study on 5623 individuals also reported a higher risk of infection among BAME individuals (positivity rate 32.1% BAME vs 18.7% White Ethnicity), while showing how the risk related to BAME increased for higher Body Mass Index (BMI) values in comparison with the White population (for BMI = 25: OR 0.96, 95% CI [0.61–1.52]; for BMI = 35: OR 2.56, 95% CI [1.63–4.03]) [39].

Other studies from the UK, using different data sources, confirm the increased risk of infection among ethnic minorities described so far. In a cross-sectional study conducted among 3018 adult patients at Sheffield Teaching Hospitals, BAME patients were significantly more likely to test positive than the White cohort (*p* = 0.026). BAME men were significantly more likely to test positive compared to both BAME women (*p* = 0.007) and White men (*p* = 0.009). Furthermore, positive BAME patients were significantly younger than White patients, with a median age of 55 years compared to 77 years (*p* < 0.001) [40]. However this study, reported in a letter to the editor, has been assessed as being of low quality according to the criteria considered.

A cross sectional study conducted among 3802 individuals in the Oxford-Royal College of General Practitioners Research Surveillance Centre reported that, compared with White people, people from Black and Asian ethnicity were at increased risk of testing positive for SARS-CoV-2 (adjusted OR 4.75, 95% CI [2.65–8.51] for Blacks and adjusted OR 1.46, 95% CI [0.94–2.29] for Asians). The prevalence rate among White people was 15.5% (388/2497) and 62.1% (36/58) among Black people [41].

A cross-sectional study conducted in east London, using primary care electronic health data from 1.2 million adult patients, found a two-fold increase in the odds of suspected COVID-19 (based on contact history and symptoms) for South Asian (OR 1.98, 95% CI [1.86–2.09]) and Black (OR 1.88, 95% CI [1.77–2.0]) adults compared with White adults (reference). In a fully adjusted analysis that included clinical factors, South Asian patients still had nearly twice the odds of suspected infection (OR 1.93, 95% CI [1.83–2.04]), while the OR for Black patients was reduced to 1.47 (95% CI [1.38–1.57]) [42]. A prospective population-based study in the UK and the USA (United States of America) confirmed that, in the general community (*N* = 2,035,395, among which 93.9% were from the UK), the hazard ratio (HR) was increased for individuals from Black, Asian, and minority ethnic backgrounds compared with White individuals (adjusted HR 2.51, 95% CI [2.18–2.89]) [43].

A retrospective cohort study carried out in University Hospitals of Leicester National Health Service (NHS) Trust UK among 4051 participants found that BAME Polymerase Chain Reaction (PCR) positivity was more common among individuals with ethnic minority backgrounds than their White counterparts (White 20.0%, South Asian 37.5%, Black 36.1%, Other 32.2%; *p* < 0.001 for all ethnic minority groups vs White). Compared to White ethnicity, South Asian, Black and Other ethnicities were more likely to test positive (adjusted OR 2.44, 95% CI [2.01–2.97], 2.56, 95% CI [1.71–3.84] and 2.53, 95% CI [1.74–3.70], respectively), as they had a larger household size (adjusted OR 1.06, 95% CI [1.02–1.11]). Belonging to an ethnic minority group was associated with PCR positivity both before and after the lockdown (adjusted OR 2.70, 95% CI [1.86–3.91] and adjusted OR 2.45, 95% CI [1.98–3.02], respectively). Moreover, when compared to the White cohort, South Asian and Black individuals were more likely to live in a deprived area (median Multiple Deprivation Index 6 vs 4, *p* < 0.001 and 6 vs 3, p < 0.001 respectively) [44, 45]. With regard to the possible correlation with social background, a socio-economic study conducted in the UK [46] reported that minority groups typically reside in parts of the country where more cases have been confirmed: Black Caribbean individuals on average reside in areas with 17% more confirmed cases per capita than White British individuals. It was reported also that they might be more at risk of community transmission due to different family and household structures. In particular, South Asian ethnic groups are more likely to live in larger households. Minority ethnic groups tend to be more likely to live in overcrowded accommodation: the proportion of households in London having more residents than rooms is 2% for White British, nearly 30% for Bangladeshis, 18% for Pakistanis and 16% for Black Africans. Finally, it is reported that Black Africans and Black Caribbeans are over-represented among key workers. In the Black African ethnic group almost 1/3 of the working-age population are employed in key worker roles and 1/5 in health and social care jobs. 37% of the UK’s doctors are foreign-born (even if only 18% of the working-age population are foreign-born), nearly 10% are from India, while 7% of nurses are Black Africans.

Conversely in a study conducted in Israel, the authors estimated the association of socio-economic status, population density, size of elderly population and ethnicity with COVID-19 morbidity and observed that Arab communities, despite their lower socioeconomic and minority status, sustained lower morbidity rates than Jewish communities, who sustained 1.68 higher morbidity rates. At any given value of population density or size of elderly population, the gap in morbidity rate between the Jewish and Arab cities is reported to persist, with morbidity rates consistently lower in Arab communities [47].

#### Healthcare workers

We found 5 data sources, all from the UK, focusing specifically on the risk of infection in healthcare workers (HCWs) during the first wave of the pandemic, most of which confirmed the disproportionate impact of COVID-19 on minority staff. A cross-sectional study conducted in April in a hospital of Birmingham among 516 HCWs, demonstrated that employees of BAME ethnicity were at significantly greater risk of seropositivity than individuals of White ethnicity (adjusted OR 1.92, 95% CI [1.14–3.23]) [48]. In a cross-sectional study conducted in a Hospital of Leicester, seroprevalence among 10,662 staff members was specifically higher in Black (21.2%) and South Asian (12.3%) compared to White staff (9.1%). Minority ethnicity was associated with seropositivity on an adjusted analysis (Black: adjusted OR 2.42, 95% CI [1.90–3.09] and South Asian: adjusted OR 1.26, 95% CI [1.07–1.49]). In particular, Black and South Asian nurses had significantly higher seropositivity rates than their White counterparts (23.9% vs 11.0% [*p* < 0.001] and 17.7% vs 11.0% [p < 0.001], respectively) [49]. Finally, the previously mentioned prospective population-based study, conducted in USA and UK [43], also showed that, among 99,795 HCWs (85.4% from UK), Black, Asian, and minority ethnic staff had an increased risk of COVID-19 (adjusted HR 1.81, 95% CI [1.45–2.24]) compared with non-Hispanic White HCWs (reference). Moreover, non-White HCWs more frequently reported reuse of or inadequate access to Personal Protective Equipment (PPE), even after adjusting for exposure to patients with COVID-19 (adjusted OR 1.49, 95% CI [1.36–1.63]) and were more likely to work in higher risk clinical settings (adjusted OR 1.13, 95% CI [1.03–1.23]). However, among 2100 staff of NHS Trust at Gateshead, the seroprevalence of BAME and White HCWs was not found to be significantly different (respectively 19.4 and 19.5%, *p* = 1.00), and the risk remained similar (BAME OR 1.03, 95% CI [0.56–1.87]) adjusted for age, gender and role within the organization [50]. Another cross-sectional study conducted among 991 staff members of County Durham and Darlington NHS Foundation Trust also found no statistically significant difference in incidence of SARS-CoV-2 RNA detection between BAME and non-BAME groups (45.1% vs. 45.3%; adjusted OR 1.08, 95% CI [0.56–2.04]), but 37% of the sample did not declare their ethnicity [51].

#### Specific vulnerable subgroups

We found a study focused on 427 pregnant women in the UK [52], which showed that 56% of women admitted to 194 hospitals with confirmed SARS-CoV-2 infection were from Black or other ethnic minority groups. The incidence per 1000 maternities was 3.5 for White women (reference), 28.4 for Blacks (rate ratio 8.1, 95% CI [6.2–10.5]), 13.9 for Asians (rate ratio 4.0, 95% CI [3.1–5.1]), 9.5 for Chinese/other (rate ratio 2.7, 95% CI [1.7–4.0]), and 6.9 for mixed ethnicities (rate ratio 2.0, 95% CI [0.9–3.8]).

A French study evaluating a population of 1216 kidney recipients between March and April 2020 showed that COVID-19–positive patients were more frequently non-Whites compared with patients who were COVID-19 negative (36.4% (24/66) patients versus 17.2% (198/1150; *p* = 0.001). The simple logistic regression analysis identifies non-White ethnicity as a factor independently associated with COVID-19 disease (adjusted OR 2.17, 95% CI [1.23–3.78]; *p* = 0.007) [53].

## Discussion

This systematic review aims at providing an overview of the risk of SARS-CoV-2 infection among migrants and ethnic minorities in the countries of the European Region of WHO. The geographical restriction to this region was chosen to limit the heterogeneity of the target population among different countries, although also countries of the European Region of WHO encompass dissimilar migration patterns and different political, cultural and economic profiles.

However, in our review we found studies only from 7 countries out of 53. In particular, 68% of the studies included were conducted in the UK: this could be due to a different sensitivity to the topic of health inequalities and possibly to a standardized data collection system that includes ethnicity. It should also be noted that all 25 studies included refer to the ‘first wave’ of the pandemic (March–June 2020), which had a different management and therefore a different impact than the following epidemic waves due to the initial lack of preparedness in the response to a rapidly evolving situation.

Taking into account that more than 2/3 of the included studies are of high quality, and only one was rated as low quality, most of them showed that MEMs were at higher risk of SARS-CoV-2 infection and over-represented in confirmed COVID-19 cases, corroborating data previously reported in the USA [54, 55] and in high-income countries in general [24]. Similarly, a greater impact on ethnic minorities had already been pointed out in previous pandemics, such as the 2009 H1N1 influenza pandemic [56, 57].

In this analysis, we report data delineating a specific set of vulnerabilities and risk factors for MEMs that could determine their over-exposure to SARS-CoV-2. The possible reasons, gathered both from the articles included in the systematic review and from other sources, are listed below.

i) The increased exposure could be due to the *over-representation of these groups among key workers and in public-facing jobs*, as suggested by several authors [29, 46]. In fact, service professions such as transport and domestic or construction work, being jobs requiring constant physical presence with the impossibility of remote working, are more exposed [58]. This would explain why, during the first wave lockdown, a smaller decrease in cases in ethnic minorities compared to the general population was reported [44], confirming the “luxury nature of stay-at-home orders” described by Huang [59]. Moreover, in some countries such as the UK, ethnic minorities are *overrepresented among healthcare workers* [46], that - together with their families - were the most exposed and affected category [60, 61]. However, some studies pointed out that, even among healthcare workers, there was a higher risk of infection specifically for minority staff [48], confirming what Rimmer previously reported [62, 63]. In particular, South Asian and Black HCWs were found to be more vulnerable by Martin [49]. This could be due to many reasons, such as the inadequate access to PPE or to the higher probability of working in higher risk clinical settings, as suggested by Nguyen [43].
ii) Another reason that could have contributed to the higher incidence of SARS-CoV-2 for some ethnic groups is their *higher risk of living in larger households and multi-generation housing,* with implications for transmission from younger to older and more vulnerable household members [22, 44]. In particular, South Asian ethnic groups seem more likely to live in *overcrowded accommodations* and larger households [46]. Such conditions were likely to make self-isolation much more challenging and to increase opportunities for within-household transmission [64]. In the ApartTogether survey report, in fact, migrants highlighted that they had more difficulties in following the measures because of the peculiar situation in which they were living [65].
iii) The geographic distribution of ethnic groups could also explain between-group inequalities in COVID-19 exposure [46]. In fact, SARS-CoV-2 transmission is known to be associated with *high population density due to increased social mixing* [66–68]. Several included studies, in fact, found a higher proportion of positive cases in urban and densely populated areas [41, 47].
iv) Moreover, as confirmed by the Swedish cross-sectional study [29], *social deprivation* has been associated with increased risk of COVID-19 [69, 70], similarly to other respiratory infections in general [71]. MEMs often have a higher socioeconomic disadvantage compared to the general population. As Kolin reported, BAME individuals had, on average, higher levels of material deprivation by Townsend score (a score that combines household overcrowding, non-home ownership, non-car ownership and unemployment), compared to those of White ethnicity [36]. Accordingly, MEMs are also potentially more likely to rely on public transport to get to work, again increasing their possible exposure to COVID-19 infection [24, 65, 72]. However, in several studies, adjusting for deprivation indexes, the risk is attenuated but still remains higher than the host population [36–38, 48].
v) Another possible influencing factor, as suggested by several authors, could be the *lower levels of language proficiency* that may hamper the access to public health information [22, 26, 29]: in some countries there has been a severely delayed translation of recommendations, safety measures and restrictions for infection prevention, especially during the initial phase of the pandemic. Conversely, in Israel, the immediate self-mobilized translation into Arabic of information material by activists and civil society could have represented a protective factor for Arab communities [47].
vi) In addition to the language barrier, there may have been other barriers in accessing information, related to the *marginality* of these groups in certain contexts, *with a negative impact on awareness of the problem and/or ability to take remedial action* [22, 24]. This lack of access to reliable information, resulting in limited tools to protect themselves, is revealed also in the WHO ApartTogether survey, especially for people living in more precarious housing situations and with a lower level of education [65].
vii) It should also be considered that *biological susceptibilities* may play a role in explaining the observed MEMs excess risk, as Raisi-Estabragh points out [33]. In fact, several studies indicate that some ethnic groups have a higher prevalence of comorbidities and cardiovascular risk factors, including obesity [73, 74]. A higher BMI has previously been reported to have an impact on the risk of infection in addition to being a predictor for poor prognosis [75]. According to Razieh, moreover, the BMI acts as an effect modifier for the increased risk of COVID-19 disproportionately between Whites and BAME [39]. However, it should be considered that the BMI also depends on the prevalence of unhealthy habits and other behaviours influenced by social determinants [26], confirming once again the role of socio-economic status and social deprivation on the higher risk of COVID-19.

In sum, most of the included studies converge towards an interpretation in terms of structural determinants of health. Studies that have probed different interpretative hypotheses, such as a possible protection given by the Bacille Calmette-Guerin vaccine (more frequent in migrants from endemic countries) [30, 76], did not find significant evidence.

Although most of the studies included show that in general the risk of infection is higher, there could be the possibility of underestimating the phenomenon. In fact, it is acknowledged that MEMs have a lower access to health services and therefore may have taken fewer SARS-CoV-2 tests, due to a lack of entitlement to free healthcare, to the previously mentioned language/cultural barriers, and possibly to a lack of trust and reliance on the health system [22, 24]. For instance, MEMs could have had a lower assistance-seeking behaviour due to inadequate health insurance or to the fear of losing their job and, especially if illegally hired or with precarious contracts, not being paid in case of sickness [77–79]. Moreover, undocumented migrants may not have accessed health services due to concerns around immigration and fear of deportation [65].

Additionally, it should be considered that during the first wave only symptomatic cases were tested for SARS-CoV-2. On one hand, the actual number of cases among MEMs might be underestimated because migrants (especially if recently arrived) are widely reported to be younger and therefore more likely to be paucisymptomatic/asymptomatic [40, 44]. On the other hand, due to their pre-existing comorbidities and therefore a higher risk of poor prognosis, MEMs may have been more likely to be admitted to hospital - and therefore tested - than the general population, with a possible overestimation [37, 64]. Nevertheless, in Reggio Emilia and in Madrid the probability of being tested was similar among immigrants and the host population [26, 30]; while two studies conducted in the UK pointed out that not only the likelihood of testing was increased, but also the likelihood of a positive test was higher among ethnic minorities who had been tested [37, 38].

However, the impact of COVID-19 on minorities does not seem homogeneous, since some ethnic groups seem to be more vulnerable than others [80]. Bearing in mind that comparisons between ethnic groups in some studies are not meaningful, perhaps because disaggregating reduces the power of the sample, many studies report that Blacks were more affected than others [38, 41], along with South Asians [42] and Latin Americans [25–28, 37]. Variations between different ethnic groups raise the possibility of ethnic-specific effects, confirming the fact that migrant populations are extremely heterogeneous [22, 33]. This could be due to behavioural responses to physical distancing measures, different among ethnic groups as a result of cultural factors, lifestyle, or religious differences. For instance, Amengual-Moreno suggests that the Asian population may have had a higher awareness due to previous affectation in their home countries, which may have led them to take measures of physical distancing and closure of their establishments before it was recommended [25].

Nonetheless, the study conducted in Israel [47] has several peculiarities due to the different composition of the population and its characteristics, despite being part of the WHO European Region. In this case, although belonging to an underprivileged, poorer minority and often living in multigenerational households, the authors found lower morbidity in Arab communities. As suggested by the authors, this could be due to the spatial Arab segregation (their peripheral location, distance from major sites of contagion and their international isolation), the lower population density, smaller families (compared to Ultra-Orthodox Jewish), and different religious behaviours. They furthermore suggest some protective factors of Israel’s Arab communities, including a highly efficient community self-organization (due to fear of no access to healthcare or stigmatization). As the authors acknowledge, however, it must be considered that the probability of being tested, which was not investigated, could play a role in explaining the different morbidity rate observed.

One issue that remains to be explored is the gender question within the ethnic minority groups: one of the cross-sectional studies in the UK found a higher likelihood of testing positive for BAME men compared to women [40], while the Italian prevalence study found a higher probability of testing positive for immigrant women, suggesting that they may have undergone a swab only when symptoms were more predictive and severe [30]. Further studies are needed to better understand the role of gender in the increased risk of infection for this vulnerable group.

### Recommendations for future research

Mixed methods quali-quantitative research will be required to fully understand the complex interplay between the various biological, social, and cultural factors underlying these findings [64]. For this purpose, it is necessary to monitor infection and disease outcomes by ethnicity and socioeconomic position. However, data allowing this disaggregation is often not available since there is relatively little routinely collected data [22]. Moreover, many countries do not have separate data collection by ethnicity but by place of birth, and this could mean that non-foreign-born individuals such as second-generation migrants are missed out, as it happens in Italy [30]. Our findings therefore corroborate the need to collect migration and ethnicity-disaggregated data advocated by several authors in order to understand the phenomenon comprehensively [81–83]. Our research also highlights that to date there are no specific studies in the literature on the risk of infection for an even more vulnerable population such as refugees, asylum seekers and migrants residing in camps or reception/detention centres. Further studies are needed to assess the final impact of COVID-19 on the health of such communities and whether they had worse outcomes compared to the host population or found more barriers to accessing health services.

### Limitations

Our systematic review has some limitations. First, grey literature and national statistics were not included, therefore some data may have been missed. We included studies published in 2020, therefore most included records concerned the first wave of the epidemic, which had some peculiar characteristics. As previously mentioned, even though our focus was the WHO European Region, most studies we found were conducted in the UK, and this is a symptom of the highly variable data collection in different countries. Lastly, not all of the included studies assessed SARS-CoV-2 infection in an objective and quantifiable manner: some considered the infection reported by patients [28, 43], while some others took into account the suspected infection based on contact history and symptoms [42].

## Conclusions

Our findings are of immediate relevance and importance to European public health. We have pinpointed gap areas to be filled in, bringing out the urgency to advocate inclusive policies and programmatic actions tailored to reach migrants and ethnic minorities. This is especially important in reducing risks of transmission and supporting the COVID-19 vaccine rollout [22, 24, 84]. Although numerous calls to consider the problem have been voiced since the beginning [11, 72], little has been done to protect migrants, refugees and ethnic minorities in the context of the pandemic.

In conclusion, our results converge to confirm the impact of structural determinants of health as modulators of risk also in the case of SARS-CoV-2 infection. Our work shows that the pandemic has widened pre-existing social health inequalities [84, 85] and that, especially since COVID-19 is a global contagious disease, there is “*No Public Health without Refugee and Migrant Health*” [9].

## Supplementary Information

**Additional file 1.** Search strategy, Detailed search strategy description.

**Additional file 2.** Additional table with detailed characteristics of the included studies.

## Data Availability

Data sharing is not applicable to this article as no datasets were generated or analysed during the current study.

## Abbreviations

MEMs: Migrants and Ethnic Minorities
SARS-CoV-2: Severe Acute Respiratory Syndrome Coronavirus-2
WHO: World Health Organization
PRISMA: Preferred Reporting Items for Systematic Reviews and Meta-Analyses
IOM: International Organization for Migration
RR: Relative Risk
OR: Odds Ratio
UK: United Kingdom
BAME: Black, Asian, and Minority Ethnic
BMI: Body Mass Index
USA: United States of America
HR: Hazard Ratio
NHS: National Health Service
PCR: Polymerase Chain Reaction
HCWs: Healthcare Workers
PPE: Personal Protective Equipment

## Footnotes

1 We referred to the migrant definition described in the IOM glossary: “An umbrella term, not defined under international law, reflecting the common lay understanding of a person who moves away from his or her place of usual residence, whether within a country or across an international border, temporarily or permanently, and for a variety of reasons. The term includes a number of well-defined legal categories of people, such as migrant workers; persons whose particular types of movements are legally defined, such as smuggled migrants; as well as those whose status or means of movement are not specifically defined under international law, such as international students”

2 We referred to refugee definition from the convention and protocol relating to the status of refugees of the United Nations High Commissioner for Refugees: (Any person who) “As a result of events occurring before 1 January 1951 and owing to well founded fear of being persecuted for reasons of race, religion, nationality, membership of a particular social group or political opinion, is outside the country of his nationality and is unable or, owing to such fear, is unwilling to avail himself of the protection of that country; or who, not having a nationality and being outside the country of his former habitual residence as a result of such events, is unable or, owing to such fear, is unwilling to return to it”

3 We referred to the ethnic minorities definition according to the European Centre for Disease Prevention and Control: “Groups of people sharing a different ‘sense of identity and common characteristics such as language, religion, tribe, nationality, race or a combination thereof’ from the majority population in the place where they live”

## References

[1] WHO 2021 COVID-19 [Internet]. [cited 2021 Jun 14]. Available from: https://covid19.who.int

[2] Johns Hopkins University COVID-19 Dashboard [Internet]. [cited 2021 Jun 14]. Available from: https://coronavirus.jhu.edu/map.html

[3] Wang ML, Behrman P, Dulin A, Baskin ML, Buscemi J, Alcaraz KI (2020). Addressing inequities in COVID-19 morbidity and mortality: research and policy recommendations. Transl Behav Med.

[4] Pūras D, de Mesquita JB, Cabal L, Maleche A, Meier BM (2020). The right to health must guide responses to COVID-19. Lancet.

[5] Wang Z, Tang K (2020). Combating COVID-19: health equity matters. Nat Med.

[6] Davies A, Basten A, Frattini C (2009). Migration: A Social Determinant of the Health of Migrants.

[7] Abubakar I, Aldridge RW, Devakumar D, Orcutt M, Burns R, Barreto ML (2018). The UCL-Lancet Commission on migration and health: the health of a world on the move. Lancet.

[8] Ingleby D, Krasnik A, Lorant V, Razum O (2012). Health inequalities and risk factors among migrants and ethnic minorities.

[9] World Health Organization. Report on the health of refugees and migrants in the WHO European Region: no public health without refugee and migrant health. 2018. ISBN 978-92-890-5384-6. https://www.euro.who.int/en/publications/html/report-on-the-health-of-refugees-and-migrants-in-the-who-european-region-no-public-health-without-refugee-and-migrant-health-2018/en/index.html.

[10] Orcutt M, Patel P, Burns R, Hiam L, Aldridge R, Devakumar D, et al. Global call to action for inclusion of migrants and refugees in the COVID-19 response. Lancet. 2020:1482–3. 10.1016/S0140-6736(20)30971-5. 10.1016/S0140-6736(20)30971-5 PMC7180034 32334651

[11] Bhopal RS. COVID-19: immense necessity and challenges in meeting the needs of minorities, especially asylum seekers and undocumented migrants. Public Health. 2020:161–2. 10.1016/j.puhe.2020.04.010. 10.1016/j.puhe.2020.04.010 PMC7158786 32325326

[12] Brandenberger J, Baauw A, Kruse A, Ritz N (2020). The global COVID-19 response must include refugees and migrants. Swiss Med Wkly.

[13] Horbach SPJM (2020). Pandemic publishing: medical journals strongly speed up their publication process for COVID-19. Quant Sci Stud.

[14] LitCovid [Internet]. [cited 2021 Mar 31]. Available from: https://www.ncbi.nlm.nih.gov/research/coronavirus/

[15] de Souza TA, da Silva PHA, da Silva Nunes AD, de Araújo II, de Oliveira Segundo VH, de Oliveira Viana Pereira DM (2020). The association between race and risk of illness and death due to COVID-19: A protocol for systematic review and meta-analysis. Medicine (Baltimore).

[16] Sze S, Pan D, Nevill CR, Gray LJ, Martin CA, Nazareth J, et al. Ethnicity and clinical outcomes in COVID-19: a systematic review and meta-analysis. EClinicalMedicine. 2020;29. 10.1016/j.eclinm.2020.100630. 10.1016/j.eclinm.2020.100630 PMC7658622 33200120

[17] Raharja A, Tamara A, Kok LT. Association Between Ethnicity and Severe COVID-19 Disease: a Systematic Review and Meta-analysis. J Racial Ethn Heal Disparities. 2020. 10.1007/s40615-020-00921-5. 10.1007/s40615-020-00921-5 PMC7659894 33180278

[18] WHO 2021 [Internet]. [cited 2021 Mar 31]. Available from: https://www.who.int/about/regions/euro/en/

[19] PRISMA [Internet]. Available from: http://www.prisma-statement.org/

[20] IOM. Glossary on Migration [Internet] 2019. Available from: https://publications.iom.int/system/files/pdf/iml_34_glossary.pdf

[21] UNHCR. Convention and Protocol relating to the Status of Refugees [Internet]. Geneva. 2010. Available from: https://www.unhcr.org/protection/basic/3b66c2aa10/convention-protocol-relating-status-refugees.html.

[22] European Centre for Disease Prevention and Control. Reducing COVID-19 transmission and strengthening vaccine uptake among migrant populations in the EU/EEA – 3 June 2021. ECDC: Stockholm; 2021.

[23] JBI’s Critical Appraisal Tools [Internet]. [cited 2021 Apr 15]. Available from: https://jbi.global/critical-appraisal-tools

[24] Hayward SE, Deal A, Cheng C, Crawshaw A, Orcutt M, Vandrevala TF (2021). Clinical outcomes and risk factors for COVID-19 among migrant populations in high-income countries: a systematic review. J Migr Heal.

[25] Guijarro C, Pérez-Fernández E, González-Piñeiro B, Meléndez V, Goyanes MJ, Renilla ME (2021). Riesgo de COVID-19 en españoles y migrantes de distintas zonas del mundo residentes en España en la primera oleada de la enfermedad. Rev Clínica Española.

[26] Jaqueti Aroca J, Molina Esteban LM, Garcia-Arata I, Garcia-Martinez J. COVID-19 in Spanish and immigrant patients in a sanitary district of Madrid. Rev Esp Quimioter Publ Of la Soc Esp Quimioter. 2020:289–91. 10.37201/req/041.2020. 10.37201/req/041.2020 PMC7374027 32434297

[27] Amengual-Moreno M, Calafat-Caules M, Carot A, Rosa Correia AR, Río-Bergé C, Rovira Plujà J, Valenzuela Pascual C, Ventura-Gabarró C. Determinantes sociales de la incidencia de la Covid-19 en Barcelona: un estudio ecológico preliminar usando datos públicos [Social determinants of the incidence of Covid-19 in Barcelona: a preliminary ecological study using public data.]. Rev Esp Salud Publica. 2020;94:e202009101. Spanish. PMC11582832 32935664

[28] Burton-Jeangros C, Duvoisin A, Lachat S, Consoli L, Fakhoury J, Jackson Y (2020). The impact of the Covid-19 pandemic and the lockdown on the health and living conditions of undocumented migrants and migrants undergoing legal status regularization. Front Public Heal.

[29] Lundkvist Å, Hanson S, Olsen B (2020). Pronounced difference in Covid-19 antibody prevalence indicates cluster transmission in Stockholm, Sweden. Infect Ecol Epidemiol.

[30] The Reggio Emilia Covid-19 working group (2020). Prevalence of SARS-CoV-2 (Covid-19) in Italians and in immigrants in an area of northern Italy (Reggio Emilia). Epidemiol Prev.

[31] Sudlow C, Gallacher J, Allen N, Beral V, Burton P, Danesh J, et al. UK biobank: an open access resource for identifying the causes of a wide range of complex diseases of middle and old age. PLoS Med. 2015;12:e1001779. 10.1371/journal.pmed.1001779 PMC4380465 25826379

[32] Woolford SJ, D’Angelo S, Curtis EM, Parsons CM, Ward KA, Dennison EM, et al. COVID-19 and associations with frailty and multimorbidity: a prospective analysis of UK Biobank participants. Aging Clin Exp Res [Internet]. 2020;32:1897–905. Available from: 10.1007/s40520-020-01653-6. 10.1007/s40520-020-01653-6 PMC7377312 32705587

[33] Raisi-Estabragh Z, McCracken C, Bethell MS, Cooper J, Cooper C, Caulfield MJ (2020). Greater risk of severe COVID-19 in Black, Asian and Minority Ethnic populations is not explained by cardiometabolic, socioeconomic or behavioural factors, or by 25(OH)-vitamin D status: study of 1326 cases from the UK Biobank. J Public Health (Oxf)..

[34] Malmström M, Sundquist J, Bajekal M, Johansson SE (1998). Indices of need and social deprivation for primary health care. Scand J Soc Med. Sweden.

[35] ReStore [Internet]. [cited 2021 Jun 14]. Available from: https://www.restore.ac.uk/geo-refer/36229dtuks00y19810000.php.

[36] Kolin DA, Kulm S, Christos PJ, Elemento O. Clinical, regional, and genetic characteristics of Covid-19 patients from UK Biobank. PLoS One [Internet]. Public Library of Science. 2020;15:e0241264. Available from: 10.1371/journal.pone.0241264. 10.1371/journal.pone.0241264 PMC7671499 33201886

[37] Niedzwiedz CL, O’Donnell CA, Jani BD, Demou E, Ho FK, Celis-Morales C, et al. Ethnic and socioeconomic differences in SARS-CoV-2 infection: prospective cohort study using UK Biobank. BMC Med. 2020;18:160. Available from: 10.1186/s12916-020-01640-8. 10.1186/s12916-020-01640-8 PMC7255908 32466757

[38] Chadeau-Hyam M, Bodinier B, Elliott J, Whitaker MD, Tzoulaki I, Vermeulen R (2020). Risk factors for positive and negative COVID-19 tests: a cautious and in-depth analysis of UK biobank data. Int J Epidemiol..

[39] Razieh C, Zaccardi F, Davies MJ, Khunti K, Yates T. Body mass index and the risk of COVID-19 across ethnic groups: analysis of UK biobank. Diabetes Obes Metab. 2020:1953–4. 10.1111/dom.14125. 10.1111/dom.14125 PMC7362044 32602268

[40] Kakkar DN, Dunphy DJ, Raza DM. Ethnicity profiles of COVID-19 admissions and outcomes. J Infect [Internet]. Elsevier; 2020;81:e110–1. Available from: 10.1016/j.jinf.2020.05.059. 10.1016/j.jinf.2020.05.059 PMC7255109 32473236

[41] de Lusignan S, Dorward J, Correa A, Jones N, Akinyemi O, Amirthalingam G (2020). Risk factors for SARS-CoV-2 among patients in the Oxford Royal College of General Practitioners Research and Surveillance Centre primary care network: a cross-sectional study. Lancet Infect Dis..

[42] Hull SA, Williams C, Ashworth M, Carvalho C, Boomla K. Prevalence of suspected COVID-19 infection in patients from ethnic minority populations: a cross-sectional study in primary care. Br J Gen Pract [Internet]. 2020;70:e696 LP-e704. Available from: http://bjgp.org/content/70/699/e696.abstract. 10.3399/bjgp20X712601 PMC7480178 32895242

[43] Nguyen LH, Drew DA, Graham MS, Joshi AD, Guo C-G, Ma W, et al. Risk of COVID-19 among front-line health-care workers and the general community: a prospective cohort study. Lancet Public Heal [Internet]. Elsevier; 2020;5:e475–83. Available from: 10.1016/S2468-2667(20)30164-X. 10.1016/S2468-2667(20)30164-X PMC7491202 32745512

[44] Martin CA, Jenkins DR, Minhas JS, Gray LJ, Tang J, Williams C, et al. Socio-demographic heterogeneity in the prevalence of COVID-19 during lockdown is associated with ethnicity and household size: Results from an observational cohort study. EClinicalMedicine [Internet]. Elsevier; 2020;25. Available from: 10.1016/j.eclinm.2020.100466. 10.1016/j.eclinm.2020.100466 PMC7366113 32840492

[45] Jordan H, Roderick P, Martin D. The Index of Multiple Deprivation 2000 and accessibility effects on health. J Epidemiol Community Health [Internet]. 2004;58:250 LP – 257. Available from: http://jech.bmj.com/content/58/3/250.abstract. 10.1136/jech.2003.013011 PMC1732697 14966241

[46] Platt L, Warwick R. COVID-19 and Ethnic Inequalities in England and Wales. Fisc Stud. 2020. 10.1111/1475-5890.12228 PMC7300623 32836536

[47] Birenbaum-Carmeli D, Chassida J. Covid-19 in Israel: socio-demographic characteristics of first wave morbidity in Jewish and Arab communities. Int J Equity Health. 2020;19:153. Available from: 10.1186/s12939-020-01269-2. 10.1186/s12939-020-01269-2 PMC7480661 32907584

[48] Shields A, Faustini SE, Perez-Toledo M, Jossi S, Aldera E, Allen JD, et al. SARS-CoV-2 seroprevalence and asymptomatic viral carriage in healthcare workers: a cross-sectional study. Thorax [Internet]. 2020;75:1089 LP – 1094. Available from: http://thorax.bmj.com/content/75/12/1089.abstract. 10.1136/thoraxjnl-2020-215414 PMC7462045 32917840

[49] Martin CA, Patel P, Goss C, Jenkins DR, Price A, Barton L, et al. Demographic and occupational determinants of anti-SARS-CoV-2 IgG seropositivity in hospital staff. J Public Health (Bangkok). 2020. Available from: 10.1093/pubmed/fdaa199. 10.1093/pubmed/fdaa199 PMC7717317 33200200

[50] Razvi S, Oliver R, Moore J, Beeby A. Exposure of hospital healthcare workers to the novel coronavirus (SARS-CoV-2). Clin Med (Northfield Il). 2020;20:e238 LP-e240. Available from: http://www.rcpjournals.org/content/20/6/e238.abstract. 10.7861/clinmed.2020-0566 PMC7687340 32962975

[51] Leeds JS, Raviprakash V, Jacques T, Scanlon N, Cundall J, Leeds CM. Risk factors for detection of SARS-CoV-2 in healthcare workers during April 2020 in a UK hospital testing programme. EClinicalMedicine. Elsevier; 2020;26. Available from: 10.1016/j.eclinm.2020.100513. 10.1016/j.eclinm.2020.100513 PMC7431176 32838245

[52] Knight M, Bunch K, Vousden N, Morris E, Simpson N, Gale C, et al. Characteristics and outcomes of pregnant women admitted to hospital with confirmed SARS-CoV-2 infection in UK: national population based cohort study. BMJ. 2020;369:m2107. 10.1136/bmj.m2107 PMC7277610 32513659

[53] Elias M, Pievani D, Randoux C, Louis K, Denis B, Delion A, et al. COVID-19 Infection in Kidney Transplant Recipients: Disease Incidence and Clinical Outcomes. J Am Soc Nephrol [Internet]. 2020;31:2413 LP – 2423. Available from: http://jasn.asnjournals.org/content/31/10/2413.abstract. 10.1681/ASN.2020050639 PMC7609004 32847984

[54] Yancy CW (2020). COVID-19 and African Americans. JAMA. United States.

[55] Webb Hooper M, Nápoles AM, Pérez-Stable EJ (2020). COVID-19 and racial/ethnic disparities. JAMA..

[56] Zhao H, Harris RJ, Ellis J, Pebody RG (2015). Ethnicity, deprivation and mortality due to 2009 pandemic influenza a(H1N1) in England during the 2009/2010 pandemic and the first post-pandemic season. Epidemiol Infect.

[57] Navaranjan D, Rosella LC, Kwong JC, Campitelli M, Crowcroft N (2014). Ethnic disparities in acquiring 2009 pandemic H1N1 influenza: a case-control study. BMC Public Health.

[58] Fasani F, Mazza J. Immigrant Key Workers: Their Contribution to Europe’s COVID-19 Response. ZA Discuss Pap No 13178. 2020. https://www.iza.org/publications/pp/155/immigrant-key-workers-their-contribution-to-europes-covid-19-response.

[59] Huang X, Lu J, Gao S, Wang S, Liu Z, Wei H. Staying at home is a privilege: evidence from fine-grained Mobile phone location data in the United States during the COVID-19 pandemic. Ann am Assoc Geogr. 2021:1–20. 10.1080/24694452.2021.1904819.

[60] Shah ASV, Wood R, Gribben C, Caldwell D, Bishop J, Weir A, et al. Risk of hospital admission with coronavirus disease 2019 in healthcare workers and their households: nationwide linkage cohort study. BMJ. 2020:371 Available from: https://www.bmj.com/content/371/bmj.m3582. 10.1136/bmj.m3582 PMC7591828 33115726

[61] Chirico F, Nucera G, Magnavita N (2020). COVID-19: Protecting Healthcare Workers is a priority. Infect Control Hosp Epidemiol.

[62] Rimmer A (2020). Covid-19: disproportionate impact on ethnic minority healthcare workers will be explored by government. BMJ.

[63] Rimmer A (2020). Covid-19: two thirds of healthcare workers who have died were from ethnic minorities. BMJ.

[64] Khunti K, Singh AK, Pareek M, Hanif W (2020). Is ethnicity linked to incidence or outcomes of covid-19?. BMJ.

[65] WHO. Preliminary Overview of Refugees and Migrants Self-Reported Impact of Covid-19 [Internet]. 2020. Available from: https://reliefweb.int/sites/reliefweb.int/files/resources/9789240017924-eng.pdf

[66] Wang K-W, Gao J, Wang H, Wu X-L, Yuan Q-F, Guo F-Y (2020). Epidemiology of 2019 novel coronavirus in Jiangsu Province, China after wartime control measures: A population-level retrospective study. Travel Med Infect Dis.

[67] Wadhera RK, Wadhera P, Gaba P, Figueroa JF, Joynt Maddox KE, Yeh RW (2020). Variation in COVID-19 hospitalizations and deaths across new York City boroughs. JAMA..

[68] Weill JA, Stigler M, Deschenes O, Springborn MR (2020). Social distancing responses to COVID-19 emergency declarations strongly differentiated by income. Proc Natl Acad Sci.

[69] Stojkoski V, Utkovski Z, Jolakoski P, Tevdovski D, Kocarev L. The socio-economic determinants of the coronavirus disease (COVID-19) pandemic. medRxiv. 2020:2020.04.15.20066068 Available from: http://medrxiv.org/content/early/2020/04/17/2020.04.15.20066068.abstract.

[70] Chung RY-N, Dong D, Li MM. Socioeconomic gradient in health and the covid-19 outbreak. BMJ. 2020;369. 10.1136/bmj.m1329. 10.1136/bmj.m1329 32238351

[71] Smith S, Morbey R, de Lusignan S, Pebody RG, Smith GE, Elliot AJ (2021). Investigating regional variation of respiratory infections in a general practice syndromic surveillance system. J Public Health (Oxf).

[72] Langellier BA. Policy recommendations to address high risk of COVID-19 among immigrants. Am J Public Health. 2020:1137–9. 10.2105/AJPH.2020.305792. 10.2105/AJPH.2020.305792 PMC7349431 32584591

[73] Falconer CL, Park MH, Croker H, Kessel AS, Saxena S, Viner RM (2014). Can the relationship between ethnicity and obesity-related behaviours among school-aged children be explained by deprivation? A cross-sectional study. BMJ Open.

[74] Leung G, Stanner S (2011). Diets of minority ethnic groups in the UK: influence on chronic disease risk and implications for prevention. Nutr Bull.

[75] Tamara A, Tahapary DL (2020). Obesity as a predictor for a poor prognosis of COVID-19: A systematic review. Diabetes Metab Syndr.

[76] Miyasaka M (2020). Is BCG vaccination causally related to reduced COVID-19 mortality?. EMBO Mol Med.

[77] Liem A, Wang C, Wariyanti Y, Latkin CA, Hall BJ. The neglected health of international migrant workers in the COVID-19 epidemic. Lancet Psychiatry. 2020:e20. 10.1016/S2215-0366(20)30076-6. 10.1016/S2215-0366(20)30076-6 PMC7129812 32085842

[78] Hargreaves S, Rustage K, Nellums LB, McAlpine A, Pocock N, Devakumar D (2019). Occupational health outcomes among international migrant workers: a systematic review and meta-analysis. Lancet Glob Heal.

[79] Giordano C (2021). Freedom or money? The dilemma of migrant live-in elderly carers in times of COVID-19. Gender Work Organ.

[80] Platt L, Warwick R. Are some ethnic groups more vulnerable to COVID-19 than others? IFS Deat Rev [Internet] 2020;1–26. Available from: https://www.ifs.org.uk/inequality/chapter/are-some-ethnic-groups-more-vulnerable-to-covid-19-than-others/

[81] Kirby T (2020). Evidence mounts on the disproportionate effect of COVID-19 on ethnic minorities. Lancet Respir Med.

[82] Pareek M, Bangash MN, Pareek N, Pan D, Sze S, Minhas JS, et al. Ethnicity and COVID-19: an urgent public health research priority. Lancet. 2020:1421–2. 10.1016/S0140-6736(20)30922-3. 10.1016/S0140-6736(20)30922-3 PMC7173801 32330427

[83] WHO. Collection and integration of data on refugee and migrant health in the WHO. 2020;110. https://www.euro.who.int/en/health-topics/health-determinants/migration-and-health/publications/2020/collection-and-integration-of-data-on-refugee-and-migrant-health-in-the-who-european-region-2020.

[84] Devakumar D, Bhopal SS, Shannon G (2020). COVID-19: the great unequaliser. J R Soc Med.

[85] Bambra C, Riordan R, Ford J, Matthews F (2020). The COVID-19 pandemic and health inequalities. J Epidemiol Community Health.

