# Supplementary material for: Risk of SARS-CoV-2 infection in migrants and ethnic minorities compared with the general population in the European WHO region during the first year of the pandemic: a systematic review
Source: BMC Public Health. 2022 Jan 20;22:143. doi: 10.1186/s12889-021-12466-1 (PMC8771174; doi:10.1186/s12889-021-12466-1)
Supplement: Supplementary file 1 — Additional file 1. Search strategy, Detailed search strategy description. [file 12889_2021_12466_MOESM1_ESM.pdf]

Systematic review: COVID-19 and migrants
Study question

Are migrants, refugees, and ethnic minorities more exposed to the risk of SARS-CoV-2 infection than the indigenous population?

Databases: Medline, Embase, Biosis, Scisearch, Esbiobase
Linguages: English, French, Spanish, Italian
Years: 2020-2021

N. Record: 1896

Search strategy

```
L1        65450 S "TRANSIENTS AND MIGRANTS"/CT OR "EMIGRANTS AND IMMIGRANTS"+NT
L2       307215 S (MIGRANT# OR IMMIGRANT# OR TRANSIENTS OR IMMIGRATION# OR
                EMIGRATION#)/TI,AB
L3        80600 S HUMAN MIGRATION+NT/CT OR MIGRATION+NT/CT
L4         6950 S (HUMAN OR INTERNATIONAL)(W)MIGRATION#/TI,AB
L5        39795 S (REFUGEE# OR INTERNALLY DISPLACED OR FORCIBLY DISPLACED OR
                INTERNATIONAL PROTECTION SEEKER# OR ASYLUM SEEKER# OR ASYLEE# OR
                SUBSIDIARY PROTECTION SEEKER# OR HUMANITARIAN PROTECTION
                SEEKER#)/TI,AB
L6          895 S UNDOCUMENTED IMMIGRANT/CT
L7          757 S (UNDOCUMENTED OR ILLEGAL OR IRREGULAR)(W)(PERSON# OR PEOPLE#
                OR INDIVIDUAL# OR POPULATION# OR PATIENT#)/TI,AB
L8          367 S (UNDOCUMENTED OR ILLEGAL OR IRREGULAR)(W)(WORKER# OR ALIEN# OR
                FOREIGNER# OR STRANGER#)/TI,AB
L9          217 S (UNAUTHORIZED OR UNAUTHORISED)(W)(PERSON# OR PEOPLE# OR
                INDIVIDUAL# OR POPULATION# OR PATIENT#)/TI,AB
L10          15 S (UNAUTHORIZED OR UNAUTHORISED)(W)(WORKER# OR ALIEN# OR
                FOREIGNER# OR STRANGER#)/TI,AB
L11      161165 S MINORITY GROUPS/CT OR MINORITY GROUP/CT OR ETHNIC GROUPS/CT
                OR ETHNIC GROUP/CT
L12      427907 S ETHNIC?(A)MINORIT?/TI,AB OR ETHNIC(W)(GROUP# OR
                COMMUNIT?)/TI,AB OR ETHNICITY/TI,AB
L13      147677 S ETHNICITY/CT
L14       83892 S RACE FACTORS/CT OR RACE/CT
L15      562240 S RACE/TI,AB OR RACES/TI,AB OR RACIAL(W)(GROUP# OR COMMUNIT? OR
                MINORIT?)/TI,AB
L16     1390187 S L1-L15
L17     9305284 S INFECTIONS+NT/CT OR INFECTION+NT/CT
L18     6845773 S (INFECTION# OR INFECTIVIT? OR INFECTIOUS)/TI,AB
L19     1140590 S (CONTAGION# OR CONTAGIOUS OR CONTAMINAT?)/TI,AB
L20      279292 S DISEASE TRANSMISSION, INFECTIOUS+NT/CT OR DISEASE
                TRANSMISSION+NT/CT
L21     2590641 S (TRANSMISSION# OR TRANSMITT?)/TI,AB
L22       30567 S VULNERABLE POPULATIONS/CT OR VULNERABLE POPULATION/CT
L23      659551 S (VULNERABLE OR VULNERABILIT?)/TI,AB
L24     5895176 S EXPOS?/TI,AB
L25    21006115 S L17-L24
L26     2026293 S RISK FACTORS/CT OR RISK FACTOR/CT OR INFECTION RISK/CT
L27    10022193 S RISK#/TI,AB
```

L28	5170167	S INCIDENCE+NT/CT OR PREVALENCE+NT/CT OR FREQUENCY/CT OR MORBIDITY+NT/CT OR EPIDEMIOLOGY/CT
L29	12684090	S (INCIDENCE# OR PREVALENCE# OR FREQUENC? OR MORBIDIT? OR EPIDEMIOLOG?)/TI,AB
L30	35938	S (HOMELESS? OR HOUSELESS? OR ROOFLESS?)/TI,AB
L31	210914	S SOCIAL CONDITIONS+NT/CT OR SOCIAL STATUS+NT/CT
L32	11838	S SOCIAL CONDITION#/TI,AB
L33	46909	S LIVING(W)(STANDARD# OR CONDITION# OR ARRANGEMENT# OR ACCOMODATION# OR SPACE#)/TI,AB
L34	514168	S HOUSING+ALL/CT
L35	169273	S HOUSING/TI,AB OR (OVERCROWD? OR OVER CROWD?)/TI,AB
L36	25778	S QUARANTINE+NT/CT OR ISOLATION+NT/CT
L37	1322065	S (ISOLATION# OR QUARANTINE# OR QUARANTAINE#)/TI,AB
L38	76935	S (CLOSE OR STRICT OR PHYSICAL OR PERSONAL OR SOCIAL)(W)CONTACT?/TI,AB
L39	20256	S (SOCIAL OR PHYSICAL OR PERSONAL)(W)DISTANC?/TI,AB
L40	32427	S PERSONAL PROTECTIVE EQUIPMENT+NT/CT
L41	43197	S PROTECT?(W)(DEVICE# OR EQUIPMENT OR PRODUCT#)/TI,AB
L42	99328	S MASKS+ALL/CT OR MASK+NT/CT
L43	44095	S (MASK# OR MOUTH PROTECTOR# OR MOUTH GUARD# OR MOUTHGUARD#)/TI,AB
L44	23	S (PROTECT? MOUTH PIECE# OR PROTECT? MOUTHPIECE#)/TI,AB
L45	15985	S GLOVES, PROTECTIVE+NT/CT OR GLOVE+NT/CT
L46	46048	S GLOVE#/TI,AB
L47	1064824	S HAND HYGIENE+ALL/CT OR HAND WASHING+ALL/CT
L48	17823	S (HAND WASHING# OR HANDWASHING#)/TI,AB
L49	21064	S (HAND HYGIENE OR HAND SANITIZATION OR HAND SANITISATION OR HAND DISINFECTION)/TI,AB
L50	13420	S "SOCIAL DETERMINANTS OF HEALTH"/CT
L51	59894	S ((HEALTH OR SOCIAL OR SOCIETAL OR SOCIOECONOMIC OR SOCIO ECONOMIC)(3A)DETERMINANT#)/TI,AB
L52	24503662	S L26-L51
L53	152579	S COVID-19/CT OR CORONAVIRUS DISEASE 2019/CT
L54	255997	S (COVID 19 OR COVID19 OR COVID 2019 OR COVID2019)/TI,AB
L55	55338	S (CORONAVIRUS DISEASE 2019 OR CORONA VIRUS DISEASE 2019)/TI,AB
L56	3100	S (CORONAVIRUS DISEASE 19 OR CORONA VIRUS DISEASE 19)/TI,AB
L57	68099	S SARS-COV-2/CT OR SEVERE ACUTE RESPIRATORY SYNDROME CORONAVIRUS 2+NT/CT
L58	3541	S (2019 NCOV OR 2019 N COV OR NCOV 2019 OR N COV 2019)/TI,AB
L59	4691	S (2019 NOVEL CORONAVIRUS OR 2019 NOVEL CORONA VIRUS OR NOVEL CORONAVIRUS 2019 OR NOVEL CORONA VIRUS 2019)/TI,AB
L60	54	S (2019 NEW CORONAVIRUS OR 2019 NEW CORONA VIRUS)/TI,AB
L61	562	S (CORONAVIRUS 19 OR CORONA VIRUS 19)/TI,AB
L62	3633	S (CORONAVIRUS 2019 OR CORONA VIRUS 2019)/TI,AB
L63	86046	S (SARS COV 2 OR SARS COV2)/TI,AB
L64	29994	S (SEVERE ACUTE RESPIRATORY SYNDROME CORONAVIRUS 2 OR SEVERE ACUTE RESPIRATORY SYNDROME CORONA VIRUS 2)/TI,AB
L65	36	S (SEVERE ACUTE RESPIRATORY SYNDROME CORONAVIRUS2 OR SEVERE ACUTE RESPIRATORY SYNDROME CORONA VIRUS2)/TI,AB
L66	648	S (SARS CORONAVIRUS 2 OR SARS CORONA VIRUS 2)/TI,AB
L67	74	S (WUHAN CORONAVIRUS OR WUHAN CORONA VIRUS OR WUHAN SEAFOOD MARKET PNEUMONIA VIRUS)/TI,AB
L68	299902	S L53-L67
L69	3549	S L16 AND L25 AND L52 AND L68
L70	3532	S L69 AND (ENGLISH OR FRENCH OR SPANISH OR ITALIAN)/LA
L71	2946	S L70 AND 2020/PY
L72	1896	DUP REM L71 (1050 DUPLICATES REMOVED)

Preprint su PubMed

Databases: medRxiv, bioRxiv, ChemRxiv, arXiv, Research Square e SSRN)

Date: 29th March 2021

Total records: 111 documenti

Search	Actions	Details	Query	Results	Time
#1	•••	⌄	Search: **PREPRINT[PT] AND (MIGRANTS OR IMMIGRANTS OR REFUGEE OR ETHNIC OR MINORITY OR RACE FACTORS OR "INTERNATIONAL MIGRATION" OR "HUMAN MIGRATION" OR ASYLUM OR ASYLEE OR FORCIBLY DISPLACED OR INTERNALLY DISPLACED OR PROTECTION SEEKER OR IRREGULAR OR ILLEGAL OR UNAUTHORIZED OR UNAUTHORISED)** "PREPRINT"[Publication Type] AND ("migrant s"[All Fields] OR "transients and migrants"[MeSH Terms] OR ("transients"[All Fields] AND "migrants"[All Fields]) OR "transients and migrants"[All Fields] OR "migrant"[All Fields] OR "migrants"[All Fields] OR ("emigrants and immigrants"[MeSH Terms] OR ("emigrants"[All Fields] AND "immigrants"[All Fields]) OR "emigrants and immigrants"[All Fields] OR "immigrant"[All Fields] OR "immigrants"[All Fields] OR "emigration and immigration"[MeSH Terms] OR ("emigration"[All Fields] AND "immigration"[All Fields]) OR "emigration and immigration"[All Fields] OR "immigration"[All Fields] OR "immigrations"[All Fields] OR "immigrant s"[All Fields] OR "immigrate"[All Fields] OR "immigrated" [All Fields] OR "immigrates"[All Fields] OR "immigrating"[All Fields]) OR ("refugee s"[All Fields] OR "refugees"[MeSH Terms] OR "refugees"[All Fields] OR "refugee"[All Fields]) OR ("ethnic groups" [MeSH Terms] OR ("ethnic"[All Fields] AND "groups"[All Fields]) OR "ethnic groups"[All Fields] OR "ethnic"[All Fields] OR "ethnical"[All Fields] OR "ethnically"[All Fields] OR "ethnicities"[All Fields] OR "ethnics"[All Fields] OR "ethnology"[MeSH Subheading] OR "ethnology"[All Fields] OR "ethnicity"[All Fields] OR "ethnology" [MeSH Terms]) OR ("minority groups"[MeSH Terms] OR ("minority" [All Fields] AND "groups"[All Fields]) OR "minority groups"[All Fields] OR "minorities"[All Fields] OR "minority"[All Fields] OR "minority s"[All Fields] OR "minors"[MeSH Terms] OR "minors"[All Fields] OR "minor" [All Fields]) OR ("race factors"[MeSH Terms] OR ("race"[All Fields] AND "factors"[All Fields]) OR "race factors"[All Fields]) OR "INTERNATIONAL MIGRATION"[All Fields] OR "HUMAN MIGRATION" [All Fields] OR ("asylum"[All Fields] OR "asylum s"[All Fields] OR "asylums"[All Fields]) OR ("asylee"[All Fields] OR "asylees"[All Fields]) OR ("FORCIBLY"[All Fields] AND ("displace"[All Fields] OR "displaced" [All Fields] OR "displacement, psychological"[MeSH Terms] OR ("displacement"[All Fields] AND "psychological"[All Fields]) OR "psychological displacement"[All Fields] OR "displacement"[All Fields] OR "displacements"[All Fields] OR "displaces"[All Fields] OR "displacing"[All Fields])) OR (("internal"[All Fields] OR "internally"[All Fields] OR "internals"[All Fields]) AND ("displace"[All Fields] OR	111	04:44:38

"displaced"[All Fields] OR "displacement, psychological"[MeSH Terms] OR ("displacement"[All Fields] AND "psychological"[All Fields]) OR "psychological displacement"[All Fields] OR "displacement"[All Fields] OR "displacements"[All Fields] OR "displaces"[All Fields] OR "displacing"[All Fields])) OR (("protect"[All Fields] OR "protected"[All Fields] OR "protecting"[All Fields] OR "protection"[All Fields] OR "protections"[All Fields] OR "protective agents"[Pharmacological Action] OR "protective agents"[MeSH Terms] OR ("protective"[All Fields] AND "agents"[All Fields]) OR "protective agents"[All Fields] OR "protectant"[All Fields] OR "protectants"[All Fields] OR "protective"[All Fields] OR "protectively"[All Fields] OR "protectiveness"[All Fields] OR "protectives"[All Fields] OR "protects"[All Fields]) AND ("seeker"[All Fields] OR "seekers"[All Fields])) OR ("irregular"[All Fields] OR "irregularities"[All Fields] OR "irregularity"[All Fields] OR "irregulars"[All Fields]) OR ("illegal"[All Fields] OR "illegality"[All Fields] OR "illegally"[All Fields] OR "illegals"[All Fields]) OR "UNAUTHORIZED"[All Fields] OR "UNAUTHORISED"[All Fields])
