# Supplementary material for: Risk of SARS-CoV-2 infection in migrants and ethnic minorities compared with the general population in the European WHO region during the first year of the pandemic: a systematic review
Source: BMC Public Health. 2022 Jan 20;22:143. doi: 10.1186/s12889-021-12466-1 (PMC8771174; doi:10.1186/s12889-021-12466-1)
Supplement: Supplementary file 2 — Additional file 2. Additional table with detailed characteristics of the included studies. [file 12889_2021_12466_MOESM2_ESM.pdf]

Citation	Location, period	Study design	Exposed population (subgroups)	Sample size	Characteristics	Not exposed population	Sample size	Characteristics	Total sample size	Objective	Outcome	Effect measures	Results	Study quality
Guijarro C., October 2020 [25]	Spain<br>February 1 April 25, 2020	Population-based cohort study	Migrants	20419 (13.4%)	**Sex**<br>Males = 9951<br>Females = 10468<br>**Age**<br>> 17 years age<br>median age = 40 years	Spaniards	131599	**Sex**<br>Males = 62588<br>Females = 69011<br>**Age**<br>> 17 years age<br>median age = 49,5 years	152018 (86.6)	To evaluate the incidence of COVID-19 in the first wave of the disease among Spaniards and migrants from different areas of the world living in Alcorcón, a city in the suburbs of Madrid (Spain) with a substantial proportion of foreign residents	Cumulative incidence of RT-PCR[b] confirmed COVID-19	Cumulative incidence,Relative Risk adjusted for age and sex	**Crude cumulative incidence rate**<br>migrants = 8.71 ,<br>Spanish population =6.51 per 1000 inhabitants (p<0.001).<br>**Adj. relative risk** (RR) people from Sub-Saharan Africa vs Spaniards: RR = 3.66 (95% CI 1.42-9.41, p = 0.007)<br>people from the Caribbean vs Spaniards: RR 6.35 (95% CI 3.83-10.55, p<0.001)<br>people from Latin America vs Spaniards:<br>RR = 6.92 (95% CI 4.49-10.67 p<0.001)	High
Jaqueti Aroca J., May 2020 [26]	Spain<br>Up to the 2nd week of April 2020	Cross-sectional	Migrants	259		Spaniads	1522		1781		Positive SARS-CoV-2 RT-PCR[b]	Positivity Rates	Risk for Covid-19 in people from Sub-Saharan Africa vs Spaniards:<br>RR 3.66 (95% CI 1.42-9.41) (p = 0.007)<br>the Caribbean vs Spaniards:<br>RR = 6.35, 95% CI (3.83-10.55) (p<0.001)<br>Latin America vs Spaniards:<br>RR 6.92, 95% CI (4.49-10.67) (p<0.001)	Medium
Amengual-Moreno M., September 2020 [27]	Spain<br>Until May 14, 2020	Ecological study	Migrants	24,5% of the total population	NA	Total population of the the neighbourhoods of Barcellona	75,5% of the total population	NA	1,600,00	To determine the relation of social determinants in the incidence of Covid-19 in the city of Barcelona	Correlation of the cumulative incidence confirmed by PCR[b] with percentage of immigration of the neighbourhood	r (Pearson linear correlation coefficient)	Statistically significant negative correlation between cumulative incidence of COVID-19 and percentage of immigration in the neighbourhood: r= -0.257; p<0.05<br>Statistically significant positive correlation between cumulative incidence of COVID-19 and percentage of Latin American immigrants in the neighbourhood: r=0.322; p<0.01	High
Burton-Jeangros C., December 2020 [28]	Switzerland<br>April – May 2020	Cross-sectional mixed-methods study	Migrants (Undocumented and recently regularized migrants)	108	Women: 85 (78.7%)<br>Men: 23 (21,3%)<br>Median age (IQR): 47.7 (16.3)<br>From Latin America: 68 (63.0%)<br>From Asia: 27 (25.0%)<br>From Non-EU/EFTA Europe: 5 (4.6%)	NA	NA	NA	108	To describe the impact of the COVID-19 crisis on the health and living circumstances of precarious migrants in Switzerland and to assess whether those undergoing legal status regularization fared better than undocumented migrants	Referred COVID-19 infection in a questionnaire	Prevalence of reported infection	Prevalence of COVID-19 infection: 12% of the interviewees reported having had infection	Medium
Lundkvist A., August 2020 [29]	Sweden<br>June 17-18, 2020	Cross-sectional study	Residents in a highly segregated low-income urban area with 98.9% of people of non-Swedish origin	90	Females: 58%<br>Males: 42%<br>Mean age: 37 years	Residents in a newly built upperand middle-class area of Stockholm with 98.4% of people of Swedish origin	123	Females: 29%<br>Males: 71%<br>Mean age: 50 years	213	To determine the prevalence rates between different areas in Stockholm, with different socio-economic conditions	Positive tests for SARS-CoV-2 antibodies	Prevalence	**Positivity**<br>Residents in the highly segregated low-income area = 30%<br>Residents in the newly built upperand middle-class area: = 4.1%<br>p<0.001	Medium
The Reggio Emilia COVID-19 Working Group, August 2020 [30]	Italy<br>March 6 – March 26, 2020	Prevalence study	Migrants (immigrant residents)	225	Males: 101<br>Females: 114 ≤ 47 years: 106 48-60 years: 66 > 60 years: 43	Italian residents in Reggio Emilia province (Italy)	2420	Males: 1294<br>Females: 1126<br>≤ 47 years: 590<br>48-60 years: 587<br>> 60 years: 1243	2635	To present the age- and sex-adjusted probability of being tested and of receiving a diagnosis of COVID-19 infection and the positive predictive value of the referral to swab in immigrants and Italians in Reggio Emilia province and to test the protective role of BCG vaccination	Positive SARS-CoV-2 PCR[b]	Prevalence, Odds Ratio adjusted for age and sex	**Probability of being positive** among those tested between immigrants and Italians: OR = 1.1 (95% CI 0.83-1.5)<br>**Proportion of tested people** among foreigners and Italians: OR = 0.93 (95% CI 0.81-1.1) The **adjusted ORs** of the probability of testing positive immigrant women vs immigrant men: OR = 2.7 (95% CI 1.4-4.9) Italian women vs italian men: OR = 0.74 (95% CI 0.62-0.88).	High

Woolford S.J., July 2020 [32]	UK<br>March 16 – June 1, 2020	Prevalence study	BAME[a]	34011		White ethnicity	468629		502640<br>**Sex**<br>Male = 226,921 (45.6%)<br>Female = 275719 (54.4%)<br>**Age** at baseline (years)= 58.0 (50.0, 63.0)	To investigated whether frailty and multimorbidity were associated with risk of hospitalisation with COVID-19.	Positive SARS-CoV-2 PCR[b]	Prevalence	Non-White individuals = 13.8% of the COVID-19 positive cohort and 7.9% of the negative one (p<0.001)	Medium
Raisi-Estabragh Z., August 2020 [33]	UK<br>March 16 – May 18, 2020	Prospective cohort study	BAME[a]	BAME ethnicity = 415<br>Mixed ethnicity = 33		White ethnicity	4068		4510<br>**Sex**<br>Men = 2201<br>Women = 2309<br>**Age** = 68.11 (± 9.23) for test positive<br>Age = 68.91 (± 8.72) for negative test	To examine whether the greater severity of coronavirus disease 2019 (COVID-19) amongst men and Black, Asian and Minority Ethnic (BAME) individuals is explained by cardiometabolic, socio-economic or behavioural factors.	COVID-19 positive test	Incidence Rates OR adjusted for sex, age, ethnicity,<br>smoking, BMI, diabetes, hypertension, high cholesterol, prior MI	**Positive COVID-19 test**<br>BAME ethnicity = 13.1% (Black ethnicity = 5.7%, Asian ethnicity = 4.5%)<br>**Risk for COVID-19 positive status** (BAME ethnicity vs White ethnicity): adj OR = 1.78, 95% CI (1.43-2.20)	High
Kolin D.A., November 2020 [36]	UK<br>From March 16, 2020	Prospective cohort study	BAME[a] (Black and Asian races)	Black race = 10181<br>Asian race = 7431		White race	374090		397064<br>**Age** (mean, SD) = 56.4 (8.1)<br>**Sex**<br>Males = 179353 (45.2%)<br>Females = 217711 (54,8%)	To analyzed the effects of clinical, regional, and genetic factors on Covid-19 positive status	Positive SARS-CoV-2 PCR[b]	Relative Risk, Odds ratio, adjusted Relative Risk for age, sex, body-mass index, systolic blood pressure, and Townsend deprivation index	**Risk of Covid-19** (vs White race): RR = 3.66 (95% CI 2.83–4.74)<br>Adj RR = 2.44 (95% CI [1.86–3.20])	High
Niedzwiedz C.L., May 2020 [37]	UK<br>March 16 – May 3, 2020	Prospective cohort study	Ethnic minorities	Mixed = 18<br>South Asian = 74<br>Black = 94<br>Chinese = 4<br>Other = 37		White ethnicity	White British = 2265<br>White Irish =82<br>White Other = 84		2658<br>Age = 40-70+	To investigate the relationship between ethnicity, socioeconomic position and the risk of having confirmed SARS-CoV-2 infection in the population-based UK Biobank study.	Positive SARS-CoV-2 microbiological tests	Relative risk adjusted for social variables	**Risk of testing positive for SARS-CoV-2 infection** (ethnic minorities vs White ethnicity) Black ethnicity: RR = 3.35 (95%CI 2.48–4.53)<br>South Asian ethnicity: RR = 2.42, (95% CI 1.75-3.36)<br>**Adjusted RR**<br>RR adj. for the country of birth = 3.13 (95% CI 2.18–4.48) RR adj. for a history of being a healthcare worker = 2.66 (95% CI 1.83–3.84)<br>RR adj. for other social factors including employment status, housing tenure and household size = 2.05 (95% CI 1.39–3.03) **Risk of testing positive for SARS-CoV-2 infection** for Asian ethnicity:<br>Pakistani group = RR 3.24 (95% CI 1.73–6.07)<br>Indian group = RR 1.98 (95% CI 1.26–3.09)	High
Chadeau-Hyam M., October 2020 [38]	UK<br>March 16 – May 18, 2020	Prospective cohort study	Ethnic minorities	Black ethnicity = 7946<br>Other ethnicity = 18633		White ethnicity	458833		488083<br>Mean age (SD) of non-tested paticiapants = 68.06 (8.10)<br>Mean age (SD) of tested paticiapants = 68.64 (8.88)<br>Female = 267716<br>Male = 220367	To identify risk factors for testing positive or negative for SARSCoV- 2 infection up to 18 May 2020, as well as those discriminating test positive vs test negative individuals using a test negative design approach	Positive SARS-CoV-2 RT-PCR[b]	Odds Ratio adjusted for demographic descriptors, social, health risk, medical, and environmental factors	**Risk of testing positive for SARS-CoV-2**<br>Black ethnicity vs White ethnicity: OR = 2.14 (95% CI 1.57-2.93)<br>Other ethnicities vs White ethnicity: OR = 1.68 (95% CI 1.29-2.18)<br>**OR adjusted** for demographic descriptors, social, health risk, medical, and environmental factors<br>Blacks vs Whites: OR = 1.11 (95% CI 1.03-1.19)<br>Other ethnicities vs Whites: OR = 1.14 (95% CI 1.06-1.23)	High
Razieh C., October 2020 [39]	UK<br>March 16 – June 14, 2020	Prevalence study	BAME[a] (minority ethnic - BME - groups - particularly South Asian - SA - and black African or Caribbean - BAC - populations)	271	Median (IQR) **age** = 64·8 (57·9, 73·2) years old **BMI** = 28·2 (25·2, 33·1) kg/m2 **Sex**<br>Women = 136 (50·2%)<br>Men = 135 (49.8%)	White ethnicity (white Europeans)	5352	Median (IQR) **age** = 71.3 (62·3, 76·1) years<br>**BMI** = 27·6 (24·8, 30·9) kg/m2<br>**Sex**<br>Women = 2650 (49·5%)<br>Men = 2702 (50.5%)	5623	1) Quantify the association of BMI with the risk of a positive test for COVID-19, stratified by ethnic group 2) Investigate whether the odds of COVID-19 in minority ethnic (BME) groups - particularly South Asian (SA) and black African or Caribbean (BAC) individuals relative to white Europeans (WEs) varied by BMI level.	Interaction between ethnicity and obesity on the risk of COVID-19 positive laboratory test	Prevalence, Odds Ratio stratified by Body Mass Index	**Positivity rate**<br>BAME = 32.1%<br>White Ethnicity = 18.7%<br>**Risk for higher Body Mass Index (BMI) values** (BAME vs White population)<br>BMI = 25: OR = 0.96 (95% CI 0.61-1.52)<br>BMI = 35: OR = 2.56 (95% CI 1.63-4.03)	Medium

Kakkar N., May 2020 [40]	UK<br>March 1 – April 25, 2020	Cross-sectional study	BAME[a]	296	Median age = 55 years	White ethnicity	2424	Median age = 77 years	3018 (298 of not known ethnicity)<br>**Sex**<br>Female = 1493<br>Male = 1499<br>Gender-unknown = 26	To investigate the potential associations between ethnicity and COVID-19	Positive COVID-19 tests	Prevalence	**Positive test** in BAME patients vs White cohort: p=0.026<br>BAME men vs BAME women: p=0.007, and vs White men: p=0.009	Low
de Lusignan S., May 2020 [41]	UK<br>January 28 – April 4, 2020	Cross-sectional study	Ethnic minorities	Asian = 152<br>Black = 58<br>Mixed, other = 81		White ethnicity	2497		3802 of which 1014 of missing ethnicity<br>**Age**<br>0–17 499 (13·1%)<br>18–39 = 666 (17·5%)<br>40–64 = 1316 (34·6%)<br>65–74 = 557 (14·7%) ≥75 =764 (20·1%)<br>**Sex**<br>Female = 2190 (57·6%)<br>Male = 1612 (42·4%)	To identify demographic and clinical risk factors for testing positive for SARS-CoV-2	Positive SARS-CoV-2 RT-PCR[b]	Odds Ratio adjusted for age sex, socioeconomic deprivation level, household size, settlement or population density, smoking status, BMI, hypertension, chronic kidney disease, diabetes, chronic heart disease, chronic respiratory disease, malignancy or immunocompromised	**Risk of testing positive for SARS-CoV-2** (Blacks vs Whites): adj. OR = 4.75 (95% CI 2.65–8.51) **Risk of testing positive for SARS-CoV-2** (Asiansvs Whites): adj. OR = 1.46 (95% CI 0.94–2.29)<br>**Prevalence rate**<br>White people = 15.5% (388/2,497)<br>Black people = 62.1% (36/58)	High
Hull A.S., October 2020 [42]	UK<br>January 1 – April 30, 2020	Cross-sectional study	Ethnic minorities	South Asian = 262323<br>Black = 154882<br>Other 79048		White ethnicity	479192		1257137 (281692 of not stated/missing ethnicity)<br>**Age, years**<br>18–49 = 932020<br>50–69 = 238339<br>≥70 1128 = 86778<br>**Sex**<br>Male = 636064<br>Female = 621073	To quantify the prevalence and time course of clinically suspected COVID-19 presenting to general practices, to report the risk of suspected COVID-19 by ethnic group, and to identify whether differences by ethnicity can be explained by clinical data in the GP record.	The diagnosis of suspected COVID-19 based on contact history and symptoms given by patients	Prevalence, Odds Ratio adjusted for age, sex, ethnicity, internal IMD 2015 quintilesb, QOF long-term conditions, BMI (kg/m^2)	**Odds of suspected COVID-1**9 (based on contact history and symptoms)<br>South Asian vs White: OR = 1.98 (95% CI 1.86-2.09) Black vs White: OR = 1.88 (95% CI [1.77 - 2.0)<br>**Adjusted analysis** South Asian patients vs White population: OR = 1.93 (95% CI 1.83-2.04)<br>Black patients: OR = 1.47 (95% CI 1.38-1.57)	High
Nguyen L.H., July 2020 [43]	UK, USA<br>March – April, 2020	Prospective cohort study	Ethnic minorities (general population; frontline healthcare workers)	**General community**<br>Hispanic or Latinx = 0.5%<br>Black = 0.6%<br>Asian = 2.2%<br>More than one or other = 2.9<br>Missing data for race or ethnic origin, prefer not to say = 1.3<br>**Front-line health-care workers**<br>Hispanic or Latinx = 1.1%<br>Black = 1.2%<br>Asian = 4.4%<br>More than one or other = 2.4<br>Missing data for race or ethnic origin, or prefer not to say = 2.7		Non-Hispanic white (general population; frontline healthcare workers)			**General community** = 2035395<br>Age = 44 (33–56)<br>Sex Male = 37.0%<br>Female = 63.0%<br>**Front-line health-care workers** = 99795<br>Age = 42 (33–53)<br>Sex Male = 17.0%<br>Female = 83.0%	To investigate the risk of testing positive for COVID-19, the risk of developing symptoms associated with SARS-CoV-2 infection, or both, among individuals in the UK and the USA, using data from a smartphone-based application	Report of a positive COVID-19 test through an app	Hazard Ratio adjusted for sex, history of diabetes, heart disease, lung disease, kidney disease, current smoking, and body-mass index	Risk for positive COVID-19 test (Hazard ratio (HR)) (individuals from Black, Asian, and minority ethnic backgrounds vs White individuals): adj. HR = 2.51 (95% CI 2.18–2.89)	High

Martin C.A., July 2020 [44]	UK<br>March 1 – April 28, 2020	Retrospective cohort study	Ethnic minorities	South Asian = 710<br>Black = 122<br>Other = 152		White Caucasian	3067		4051<br>**Age** (years), median (IQR) = 64 (45 78)<br>**Gender**<br>Female = 1979 (48.9%)<br>Male =2072 (51.2%)	To investigate the factors associated with prevalent COVID-19 among hospital attenders, and secondly to establish whether temporal changes in the proportion of positive test results before and after institution of lockdown measures differ by ethnicity	Positive SARS-CoV-2 PCR[b] before/after lockdown	Positivity Rates, Odds Ratio adjusted for age, gender, ethnicity, estimated household size	**Positivity rate**<br>White = 20.0%<br>South Asian = 37.5% Black = 36.1% Other = 32.2% p<0.001 for all ethnic minority groups vs White<br>**Positivity risk**<br>South Asian: adj. OR = 2.44 (95% CI 2.01-2.97)<br>Black: adj. OR = 2.56 (95% CI 1.71-3.84)<br>Other: adj. OR = 2.53 (95% CI 1.74-3.70)<br>**PCR positivity before the lockdown** (other ethnicities vs White): adj. OR = 2.70 (95% CI 1.86-3.91)<br>**PCR positivity after the lockdown** (other ethnicities vs White): adj. OR = 2.45 (95% CI 1.98-3.02)	High
Platt L., June 2020 [46]	UK<br>Up to May 2020	Ecological study	Ethnic minorities (White other, Indian, Pakistani, Bangladeshi, black African and black Caribbean)	NA		White British majority	NA		NA	To investigate the extent to which different ethnic groups have been more or less affected by the immediate impact of the COVID-19 crisis, both in terms of exposure to infection and health risks, including mortality, and in relation to their exposure to economic impacts	Predicted number of lab-confirmed COVID-19 cases per 100,000 of group population		Black Caribbean individuals on average reside in areas with 17% more confirmed cases per capita than White British individuals.<br>Proportion of households in London having more residents than rooms<br>White British = 2%<br>Bangladeshis = 30%<br>Pakistanis = 18% and Black Africans = 16%<br>Employment<br>1/3 of the Black African ethnic group are employed in key worker role<br>1/5 in health and social care jobs<br>37% of the UK's doctors are foreign-born (nearly 10% are from India, while 7% of nurses are Black Africans)	Medium
Birenbaum-Carmeli D., September 2020 [47]	Israel<br>February 23 – June 2, 2020	Cross-sectional study	Ethnic minorities (Arab communities)			Jewish communities				To explore the extent to which factors such as socio-economic status (SES), population density, size of elderly population and minority status are associated with morbidity rates in Israel's residential communities	Number of confirmed COVID-19 cases in each community	Morbidity rates	Jewish communities suffered 1.68 higher morbidity rates compared to Arab communities (b = 1.68, β = 0.437).<br>The descrpetive statisitics suggest:<br>1. Inverse association between a community's SES and its morbidity rate<br>2. Positive association between population density and morbidity<br>3. Inverse relation between percentage of elderly population and morbidity.	High
Shields A., September 2020 [48]	UK<br>April 24-25, 2020	Cross-sectional study	BAME[a] (Healthcare workers)	Asian British Bangladeshi = 2 (0.4%)<br>Asian British Indian = 25 (4.8%)<br>Asian British Pakistani = 11 (2.1%)<br>Asian British other/not stated = 18 (3.5%)<br>Asian British Pakistani = 11 (2.1%)<br>Black African = 10 (1.9%)<br>Black British = 17 (3.3%)<br>Filipino 21 (4.1%)<br>Mixed heritage= 10 (1.9%)<br>Not disclosed = 74 (14.3%)		White British (Healthcare workers)	White Irish = 5 (1.0%)<br>White other = 25 (4.8%)<br>White British = 285 (55.2%)		516<br>**Age** (years) = 42 (30–51)<br>**Sex**<br>Male = 128 (24.8%)<br>Female = 388 (75.2%)	To determine the rates of asymptomatic viral carriage and seroprevalence of SARS-CoV-2 antibodies in healthcare workers	Positive tests for SARS-CoV-2 antibodies	Prevalence, Odds Ratio adjusted forage, sex, ethnicity, Index of Multiple Deprivation score and department in which participants worked	**Risk of seropositivity** (employees of BAME ethnicity vs employees of White ethnicity): adj. OR =1.92 (95% CI 1.14-3.23)	High

Martin C.A., November 2020 [49]	UK<br>May 2020	Cross-sectional study	Ethnic minorities (Hospital staff members)	South Asian = 2494 (23.4%)<br>Black = 553 (5.2%)<br>Other= 655 (6.1%)	**Age** (years), median (IQR)<br>South Asian = 41 (31–50)<br>Black = 42 (32–49)<br>Other= 42 (33–49)<br>**Sex**, n(%<br>South Asian: Female = 1817 (72.9%) Male = 677 (27.2%)<br>Black: Female = 447 (80.8%) Male = 106 (19.2%)<br>Other: Female = 443 (67.7%) Male = 212 (32.4%)	White ethnicity (Hospital staff members)	6960 (65.3%)	**Age** (years), median (IQR) = 46 (34–55)<br>**Sex**, n(%)<br>Female = 5796 (83.3%)<br>Male = 1164 (16.7%)	10662	To examine the seroprevalence of anti-SARS-CoV-2 IgG amongst hospital staff employed at University Hospitals of Leicester (UHL) NHS Trust	Positive tests for SARS-CoV-2 antibodies	Prevalence, Odds Ratio adjusted for age, sex, ethiciy, occupation, speciality, path/Micro), IMD quintile, population density of output area, reason for absence from work	**Seroprevalence**<br>White = 9.1%<br>Black = 21.2%)<br>South Asian = 12.3% **Seropositivity** (staff of minority ethnicity vs staff of White ethnicity)<br>Black: adj. OR = 2.42 (95% CI 1.90–3.09)<br>South Asian: adj. OR = 1.26 (95% CI 1.07–1.49)<br>**Seropositivity rates**<br>Black nurses = 23.9% vs 11.0% of White nurses (p< 0.001)<br>South Asian nurse = 17.7% vs 11.0% of White nurses (p< 0.001)	High
Razvi S., November 2020 [50]	UK<br>May 28 – June 8, 2020	Cross-sectional study	BAME[a] (Healthcare workers)	72 (3.4%)		White ethnicity (Healthcare workers)	2028 (96.6%)		2100 members of staff<br>2521 samples<br>Mean **age** (SD) = 42.5 (12.6)<br>**Sex**<br>Female = 2317 (91.9%)<br>Male = 204 (8.1%)	To ascertain the prevalence of antibody positivity in HCWs and, in addition, determine if specific groups such as men, frontline staff and BAME HCWs may have had more exposure than others	Positive tests for SARS-CoV-2 antibodies	Prevalence, Odds ratio adjusted for age, gender and role within the organization	**Seroprevalence** for SARS-CoV-2 antibody<br>BAME = 19.4% White = 19.5% (p=1.00)<br>Risk of positive test (BAME vs White): adj. OR = 1.03 (95% CI 0.56–1.87)	High
Leeds J.S., August 2020 [51]	UK<br>April 1-28, 2020	Cohort study	BAME[a] (Healthcare workers)	Indian subcontinent = 22<br>Chinese Asian = 4<br>Other Asian = 9<br>Mid East = 2<br>African = 5<br>Other ethnic group = 7<br>Not stated/declined = 370		White ethnicity (Healthcare workers)	572		991<br>**Age**<br>≤ 24 = 62<br>25 - 34 = 238<br>35 - 44 = 218<br>45 - 54 = 296<br>55 - 64 = 162<br>≥ 65 = 15<br>**Sex**<br>Male = 145<br>Female = 846	To report the results of the staff testing programme in a large acute NHS Trust in the UK and examine the characteristics of affected healthcare workers including ethnicity	Positive SARS-CoV-2 RT-PCR[b]	Incidence Rates, Odds Ratio adjusted for age, male sex, acute frontline worker, BAME status	**Incidence of SARS-CoV-2**<br>BAME = 45.1%<br>non-BAME = 45.3%<br>**Risk of positive test**<br>Adj. OR = 1.08 (95% CI 0.56 – 2.04)	High
Knight M., June 2020 [52]	UK<br>March 1 – April 14, 2020	Prospective population-based cohort study	Ethnic minorities (pregnant women)	Asian = 103 (25%)<br>Black = 90 (22%)<br>Chinese/other = 30 (7%)<br>Mixed = 10 (2%)<br>Missing data = 10		White ethnicity (pregnant women)	184		427<br>**Age**<br>< 20 = 4 (1%)<br>20-34 = 248 (58%)<br>≥35 = 175 (41%)	To describe a national cohort of pregnant women admitted to hospital with SARS-CoV-2 infection,identify factors associated with infection, and describe outcomes, including transmission of infection, for mothers and infants	Cumulative incidence of PCR[b] confirmed COVID-19	Incidence Rates, Rate Ratio	**Incidence of SARS-CoV-2** (per 1000 maternities) White = 3.5 Blacks = 28.4<br>Asians = 13.9<br>Chinese/other = 28.4<br>Mixed ethnicities = 9.5<br>**Rate ratio** (BAME women vs white women)<br>Blacks: rate ratio = 8.1 (95% CI 6.2-10.5)<br>Asians: rate ratio = 4.0 (95% CI 3.1-5.1)<br>Chinese/other: rate ratio = 2.7 (95% CI 1.7-4.0) Mixed ethnicities: rate ratio = 2.0, (95% CI 0.9-3.8).	Medium
Elias M., October 2020 [53]	France<br>March 1 – April 30, 2020	Prospective cohort study	Ethnic minorities (Kidney recipients)	222		White ethnicity (Kidney recipients)	994		1216<br>**Age** = 54.1 ± 13.4<br>**Sex**<br>Male = 777<br>Female = 439	To evaluate the prevalence of COVID-19 disease in a nonselected population of kidney recipients, to detect modifiable and nonmodifiable determinants associated with the susceptibility and severity of COVID-19 infection	Positive SARS-CoV-2 PCR[b]	Odds Ratio adjusted for age, sex, ethnicity, donor type, time on dialysis before graft, time from transplant to evaluation, serum creatinine, obesity, diabetes, hypertension, asthma and chronic pulmonary disease, cardiovascular disease, and prednisone treatment	Non-White COVID-19–positive patients = 36.4% (24/66)<br>Non-White COVID-19 negative patients = 17.2% (198/1150) p=0.001<br>**Association of ethnicity with with COVID-19 disease** (non-White versus white) Adj. OR = 2.17 (95% CI 1.23-3.78) (p=0.007)	High
